# Product-stabilized filamentation by human glutamine synthetase allosterically tunes metabolic activity

**DOI:** 10.1101/2025.07.04.663231

**Published:** 2025-07-06

**Authors:** Eric Greene, Richard Muniz, Hiroki Yamamura, Samuel E. Hoff, Priyanka Bajaj, D. John Lee, Erin M. Thompson, Angelika Arada, Gyun Min Lee, Massimiliano Bonomi, Justin M. Kollman, James S Fraser

**Affiliations:** 1 - Department of Bioengineering and Therapeutic Sciences, University of California San Francisco, San Francisco, CA; 2 - Department of Biochemistry. University of Washington, Seattle WA; 3 - Institut Pasteur, Université Paris Cité, CNRS UMR 3528, Computational Structural Biology Unit, Paris, France; 4 - Department of Biological Sciences, KAIST, Daejeon, 34141 Republic of Korea

## Abstract

To maintain metabolic homeostasis, enzymes must adapt to fluctuating nutrient levels through mechanisms beyond gene expression. Here, we demonstrate that human glutamine synthetase (GS) can reversibly polymerize into filaments aided by a composite binding site formed at the filament interface by the product, glutamine. Time-resolved cryo-electron microscopy (cryo-EM) confirms that glutamine binding stabilizes these filaments, which in turn exhibit reduced catalytic specificity for ammonia at physiological concentrations. This inhibition appears induced by a conformational change that remodulates the active site loop ensemble gating substrate entry. Metadynamics ensemble refinement revealed >10 Å conformational range for the active site loop and that the loop is stabilized by transient contacts. This disorder is significant, as we show that the transient contacts which stabilize this loop in a closed conformation are essential for catalysis both *in vitro* and in cells. We propose that GS filament formation constitutes a negative-feedback mechanism, directly linking product concentration to the structural and functional remodeling of the enzyme.

## Introduction

To support the metabolic needs of a cell, enzymes must be regulated to adapt to changing nutrient conditions (Atkinson 1965). Because these changes often occur faster than the rates of transcription/translation or degradation, this regulation is conferred allosterically by downstream metabolites or posttranslational modifications, or orthosterically via substrate availability and/or product inhibition (Mathy and Kortemme 2023). In metabolism, one common allosteric mechanism is feedback inhibition whereby downstream metabolites alter the enzymatic activity of upstream processes, typically at rate-limiting or committed metabolic nodes (Gerhart and Pardee 1962). In classical models of allostery, this regulation is conferred through the binding of an effector molecule at a site other than the protein’s active site, known as the allosteric site (Cui and Karplus 2008). Allosteric ligand binding subsequently induces or stabilizes a conformational change in the protein that can manifest at the tertiary, quaternary, and/or quinary (e.g. filamentous or other supramolecular states) level leading to the activity change observed (Hvorecny and Kollman 2023).

While many well-studied metabolic enzymes have evolved dedicated small molecule or protein allosteric binding sites as a primary means of regulation, an increasing number of soluble metabolic enzymes have been observed to adopt supramolecular, filamentous quinary structures as a mechanism for allosteric regulation (Noree et al. 2019; Park and Horton 2019). Filament formation can confer multiple distinctive mechanisms of enzyme regulation including repression and activation, providing an alternative to synthesis/degradation to rapidly alter the balance of enzyme activity within the cell (Lynch, Kollman, and Webb 2020; Hvorecny and Kollman 2023). Indeed, more classic small molecule binding sites can regulate enzyme filamentation and the combination of these two modes represents a rich expansion of classical allostery, integrating structural and functional changes at the level of protein assembly.

Here, we examine how glutamine metabolism is controlled by different potential allosteric mechanisms. Glutamine levels must be critically regulated, as it uniquely serves as both an anaplerotic donor (carbon source) and a nitrogen donor in key metabolic processes (Tecson et al. 2025; Fung, Ryu, and Thompson 2025). In addition, it is, obviously, a building block in protein biosynthesis. Glutamine synthetase (GS) is an ancient enzyme that catalyzes the ATP-dependent condensation of glutamate and ammonia to glutamine (Eisenberg et al. 2000; de Carvalho Fernandes, Turchetto-Zolet, and Pereira Passaglia 2022; Tecson et al. 2025). Glutamine is the most abundant amino acid in blood, where a majority originates from *de novo* synthesis by GS (Tecson et al. 2025; Fung, Ryu, and Thompson 2025; Castegna and Menga 2018). Moreover, ammonia generated through catabolic processes is detoxified through GS-catalyzed glutamine formation. The ammonia detoxification is critically important in the brain, where loss of function GS variants cause severe developmental defects (Spodenkiewicz et al. 2016). Regulation of human GS has so far been largely described at the transcriptional or protein degradation levels (Eisenberg et al. 2000; Rigual et al. 2025; Castegna and Menga 2018) where GS expression is restricted to certain cell types and tissues and expressed GS can be degraded by the ubiquitin proteasome system in a glutamine dependent manner (Zhao et al. 2025; T. Van Nguyen et al. 2016; Thang Van Nguyen et al. 2017; Thang Van Nguyen 2021). However, regulation via these processes would require hours to achieve appreciable changes in intracellular GS concentrations. By contrast, multiple allosteric and feedback mechanisms have been described for bacterial GS homologs, including by filamentation in both *E. coli* (Eisenberg et al. 2000; Huang et al. 2022) and *S. cerevisiae* (Petrovska et al. 2014; He et al. 2009). These observations led us to ask how human GS activity could be regulated on the timescales required to adapt to dynamic changes in nutrient availability.

Here, we used a combination of cryo-electron microscopy (cryoEM), enzymology, and cell proliferation assays, to discover a regulatory mechanism that incorporates both feedback inhibition and filamentation. We find that human GS forms filamentous assemblies that are stabilized by its own reaction product, glutamine. Human GS filaments display lower catalytic efficiency for ammonia, which we attribute to changes in protein flexibility surrounding the active site. We probe the importance of this change in protein dynamics with mutagenesis using enzyme activity *in vitro* and in HEK293 cells. Our findings support a model where product-stabilized GS filaments serve as an allosteric feedback mechanism, regulating activity through modulation of the enzyme’s conformational landscape.

## Results:

### Identification of heterogeneous oligomeric assemblies of human GS

**Figure 1.**
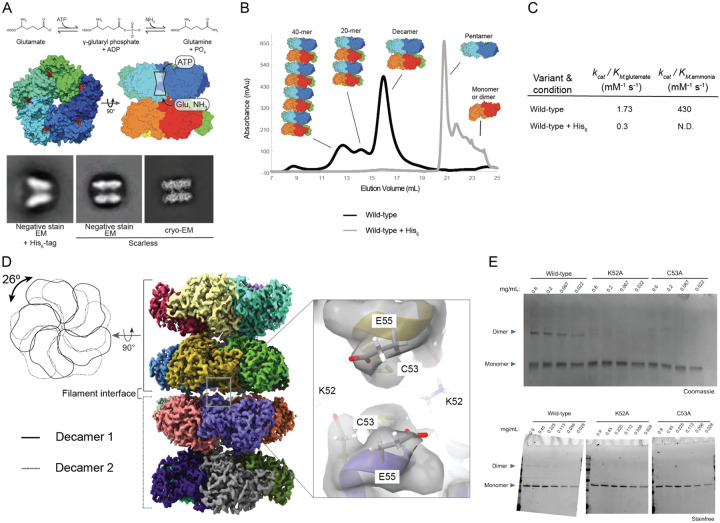
Human Glutamine Synthetase Forms Filaments. A) At top, reaction mechanism of glutamine synthetase. Middle: surface representation of PDB:2QC8 ADP + Methionine sulfoximine (MSO) bound x-ray crystal structure of human glutamine synthetase. Bottom: N-terminal His_6_-tag perturbs structure of human glutamine synthetase (GS) as observed by 2D classification of negative stain and cryoEM data. B) Size-exclusion chromatography reveals His_6_-tag dramatically alters the oligomeric state of human GS and scarless GS can form higher order oligomers. C) His_6_-tagged GS displays strongly altered steady-state kinetic constants when compared to scarless GS. N.D. = not defined as His_6_-tagged GS also display inefficient coupling of ATP hydrolysis with glutamine formation and thus *K*_*M,ammonia*_ could not be defined for this enzyme. D) cryoEM reconstruction of apo-GS filament form. Decamers are rotated relative to each other by ~26 degrees. The minimal five-fold filament interface formed consists of K52, C53, and E55 of adjacent monomers from different decamers. No obvious connecting cryoEM density is observed in the apo map. E) Bifunctional crosslinking with Bis-sulfosuccinimidyl glutarate (BSG; top) or bis-maleimoethane (BMOE; bottom) to covalently attached adjacent primary amines (K52) or cysteines (C53) respectively followed by SDS-PAGE reveals crosslinked species only in the wild-type GS protein sample, but not K52A or C53A indicating presence and disruption of filament formation respectively.

To define regulatory mechanisms of GS, we first used cryoEM to examine the conformational landscape of GS. Our initial attempts at *in vitro* characterization of GS using N-terminally His_6_-tagged constructs were met with low molecular weight complex compositions by size-exclusion chromatography (SEC) and we observed rare, decameric 2D averages by negative-stain electron microscopy (Figure 1A, B). However, most particles and 2D classes were distorted and this preparation did not result in a large population of the canonical decameric form observed in the X-ray structure of the identical GS construct (Krajewski et al. 2008). However, scarless preparations of GS, either through N-terminal His_6_-SUMO-tag and Ulp1 cleavage during purification or C-terminal Intein-CBD-His_6_ tag and DTT facilitated intein self-cleavage yielded pure enzyme with both decameric and higher molecular weight species observed by SEC (Figure 1A, B). In addition to scarless preparations yielding canonical forms of GS, we found a >5-fold higher *k*_*cat*_/*K*_*M,glutamate*_ for scarless GS, and that the His_6_-tagged counterpart has a decoupled reaction whose ADP-release was insensitive to ammonia concentration (Figure 1C, Supplementary Figure 1). These data demonstrate a significant structural and functional impact of N-terminal His_6_ tags on GS in addition to the presence of supramolecular architectures.

To investigate how differences in oligomeric state might impact the structure and function of GS, we characterized the decamer oligomeric fraction isolated by SEC by cryoEM. We observed GS to be present in decameric form, but also a resolvable population of short filament assemblies of repeating decameric units. These higher order assemblies are consistent with dynamic exchange corresponding to the distribution of SEC peaks with estimated molecular weights of ~0.3 – 1.5 MDa (Figure 1B). We isolated the 2-decameric/di-decameric particles from the decameric particles using 2D classification and non-heterogeneous refinement and reconstructed a high resolution map with FSC 0.143 of 2.38 Å (Supplementary Figure 2).

The filament was composed of two decamers (“2-decamer”) bound to each other in a ‘head-to-head’ configuration with one decamer rotated ~26° to the next (Figure 1D). The decamer-decamer (filament) interface is five-fold symmetric and made up of residues K52, C53, and E55 contributed by both monomeric subunits across the interface. Interestingly, K52 also participates in a binding interface between GS and bestrophilin2 ion channel (Owji et al. 2022). We observed little to no density at the interface despite the stable relative orientation of both decamers to each other (Figure 1D). These data suggest that the formation of the interface is concentration dependent and driven primarily by electrostatic interactions. Alternatively, the interface could be largely solvent-mediated or be connected by a ligand(s) that occupy only some of the 5 symmetry related interfaces. In both scenarios, any signal is likely to be averaged out and experiments with symmetry expansion and focused classification did not yield any convincing density.

To test the importance of the interface, we used homo-bifunctional crosslinking to probe the contributions of individual contacts. The 2-decameric map suggests that only one pair of lysine residues (K52:K52) and one pair of cysteine residues (C53:C53) are surface exposed and within <10Å across two monomers. To ensure a stringent selection for only K52 and C53 we used either the 7.7Å spacer-arm bifunctional NHS-ester crosslinker (bis(sulfosuccinimidyl) glutarate or BSG) or the 8.0Å spacer-arm biofunctional maleimide crosslinker (bismaleimidoethane or BMOE). Crosslinking of two monomers by either crosslinker was observed in the wild-type enzyme and more prevalent at higher enzyme concentrations (Figure 1E). However, mutation of either K52 or C53 to alanine ablated the crosslinking reactivity and did not support filament formation as assessed by negative stain EM (Supplementary Figure 3). Together these data support the 2-decameric filament interface assignment whose formation is concentration dependent and driven primarily by electrostatic interactions.

### Glutamine stabilizes larger GS filaments

**Figure 2:**
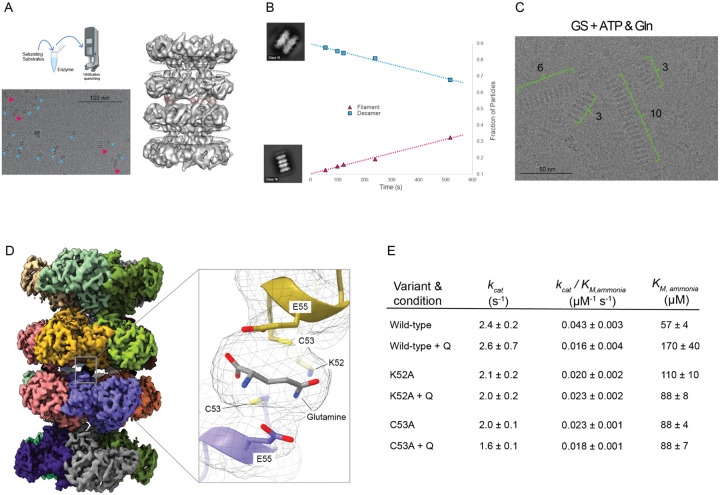
Glutamine binds to GS filaments at the interface. A) Establishment of steady state conditions prior to vitrification with GS at low concentration (0.15 mg/mL) revealed presence of 2-decameric species (red arrows) in addition to decameric species (blue arrows) during cryoEM grid screening. Initial reconstruction of the turnover 2-decameric map demonstrated clear density connecting decamers (red dotted circle) not previously observed in the apo-filament map and loss of E305-loop density (black dotted circle). B) Time-resolved cryoEM demonstrates linear relationship between glutamine and filament formation assessed by independently processing five datasets and quantifying the fraction of decamer and filament present (see Methods) and plotted against the vitrification quench time of the reaction. C) cryoEM screening data demonstrating robust filament seeding from addition of ATP and glutamine to human GS prior to vitrification. D) Turnover-filament 3D reconstruction demonstrates similar overall architecture to apo-filament map (left) but with clear density between decamers with glutamine modeled which forms hydrogen bonds with K52, C53, and E55 (right). E) Steady-state ammonia kinetic constants for wild-type, K52A, and C53A with and without 10 mM glutamine added to reaction to stimulate filament formation. Errors were calculated as S.E.M. from global fitting and were propagated through each Michaelis-Menten parameter (Equations 2–4; *n* = 8 replicates) see Methods.

The interface defined by the 2-decameric assembly and the presence of higher molecular weight species in the SEC trace suggested that larger filaments of GS can readily form. Next, we wanted to understand what functional consequence may be conferred by filamentation in human GS. We asked if GS filament formation could be retained under turnover conditions and impact the catalytic function of GS. CryoEM is an ideal tool to investigate structural changes to macromolecules under turnover conditions because substrates can be added to the enzyme immediately before vitrification freeze-quenching to preserve the sample in a near-native context (Figure 2A, Supplementary Figure 4). Turnover conditions were induced by adding saturating concentrations of substrates immediately prior to vitrification, with reaction times totalling between 49 and 59s initiated by manual mixing prior to blotting. Our initial grid screening of GS under turnover conditions identified filaments at lower GS concentrations (0.1 mg/mL) than we observed for apo GS. Initial reconstruction of these screening data revealed two new structural features in the GS filament: defined EM density between both decamers and missing density for the active site loop region (Figure 2A). Given that the only sample preparation differences between the apo-GS datasets and the turnover conditions was the addition of substrates that are converted to products prior to vitrification, we hypothesized that one or multiple substrates (ATP, glutamate, and ammonium) or products (glutamine, PO_4_, and ADP) may be responsible for this additional density. However, at this initial low resolution we could not assign a compound at this interface nor understand if an interface-bound compound had any conformational impact on active site geometry.

To discriminate whether the additional filament density corresponded to product or substrate, we performed a time-resolved (tr-)cryoEM experiment. We collected five turnover cryoEM datasets that differed only in the vitrification-quench time of the steady-state reaction with reaction times between 57s and 517s corresponding to high substrate and high product respectively (see methods for details) at a fixed concentration of enzyme (0.3 mg/mL). To understand if there was a trend in the particle populations between the decamer/filament compositions as a function of reaction time, we compared the number of particles in decameric versus filamentous states by 2D classification (Supplementary Figure 5). The same template picking parameters were applied for each tr-cryoEM dataset and 2D classification was performed twice; once with each time-point dataset processed independently and once where all particles were combined and the dataset identity only revealed after 2D classification particle classes were picked (Supplementary Figure 6). We then normalized decamer and filament particle picks to the total number of picked particles (excluding 2D classes that could not be assigned either a decamer or filament) and plotted these as a function of reaction time (Figure 2B). The positive linear correlation between fraction of particles in a filament-like assembly and reaction time suggests that a product of the forward reaction of GS acts to stabilize the filament assembly. Subsequent screening with high concentrations of glutamine revealed large >4 decameric unit filament assemblies (Figure 2C), suggesting that product formation has a positive effect on filament assembly.

To define how products stabilized the GS filament assembly, we next determined a high resolution structure of the filaments imaged under turnover conditions. We performed an additional cryoEM experiment with increased GS concentration (1.5 mg/mL), to bias our particle populations towards filamentous assemblies, and vitrified at 57s, a reaction time that should yield a high concentration of product due to the higher enzyme concentration than previous experiments. The resulting 3D reconstruction of the 2-decameric species had an FSC 0.143 of 2.2Å (Supplementary Figure 7A).

Inspection of the ‘filament assembly’ interface between the two decamers revealed a discrete density that was absent from the apo-filament reconstruction (Figure 2D). The density is too small for a nucleotide (ATP or ADP) and too large for inorganic phosphate (Supplementary Figure 7B). Given the association with product formation, we modeled glutamine into this density in a conformation consistent with forming hydrogen bonds that link the filament interface residues and the bound product. The two-fold symmetry at this interface and the averaging inherent in our data processing results in density that likely reflects contributions from glutamine in two orientations. In addition, we note two potential, and not mutually exclusive, reasons we were able to observe filaments in the apo enzyme. First, we used high concentrations of GS, which likely shifts the equilibrium towards the filament form even in the absence of glutamine. Second, there may be a small, unresolved, amount of glutamine that persists during purification that occupies only a minor fraction of interfaces per di-decamer. As discussed above, such contributions would be averaged out during map refinement in the scheme we have used here. Taken together, these data support a model wherein glutamine synthetase filamentation generates a composite binding site for glutamine at the filament interface, which is located over 10Å from the active site.

### Glutamine stabilized filaments have lower catalytic efficiency for ammonia

We next examined the functional consequences of GS filamentation on enzymatic activity. To assess steady-state kinetic differences between decameric and filamentous compositions of GS, we assayed different isotropic peak fractions resolved by SEC immediately after separation. We found k_cat_ and k_cat_/K_M_ for all substrates were completely in agreement between the decamer and 2-decamer fractions at 0.01 mg/mL for wild-type, K52A, and C53A (Supplementary Figure 8). However, this enzyme concentration is 30-fold dilute compared to typical cryoEM conditions and the 2-decameric fraction significantly decomposed into decamer, as assessed by mass photometry (Supplementary Figure 9A). Upon increasing enzyme concentration to 0.1 mg/mL, which is within the range of our cryoEM experiments (0.3 mg/mL for all datasets except where indicated) and shown to support filamentation by chemical crosslinking (Figure 1D), we uncovered a subtle, 2-fold K_M, ammonia_ defect for the 2-decamer fraction compared to the decamer fraction for wild-type GS (Supplementary Figure 9B).

To probe this defect in K_M_ further, we took the decameric fraction at 0.1 mg/mL and repeated steady-state assays in the presence or absence of the glutamine concentration used to stabilize filamentation by CryoEM (Supplementary Figure 10). The presence of glutamine increased *K*_*M, ammonia*_ three-fold for WT, but had smaller effects for the mutations at the interface that directly interact with the ligand (Figure 2E). Given the incomplete filament formation observed in our cryoEM experiments, with a maximum filament formation of ~50% at 0.3 mg/mL concentration (Supplementary Figure 6), the effective *K*_*M, ammonia*_ defect upon filamentation is likely at least 2-fold higher than measured. The range of *K*_*M, ammonia*_ values reported here are near the ammonia concentration estimates reported in the blood and liver under normal physiological conditions (<50 μM) and lower than hyperammonemic conditions (0.2 – 1 mM) (Albe, Butler, and Wright 1990; Dasarathy et al. 2017).

### The filament state impacts conformational variability of E305-loop

**Figure 3:**
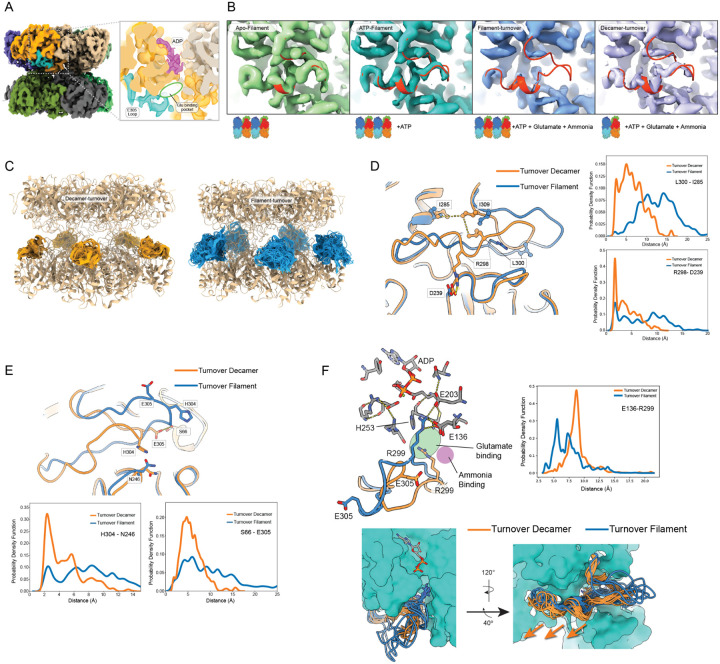
Variable E305-loop density and conformations in human glutamine synthetase. A) Turnover-decamer cryoEM density map is colored based on monomer except for one E-305 loop with is colored cyan and ADP density that is colored purple demonstrating how the E305-loop closes over the active site to enclose the glutamate and ammonia binding site. B) CryoEM E305-loop density representations for the apo-filament, ATP-filament, turnover-filament, and turnover-decamer maps with 2QC8 docked into each map shown in red. The contour is chosen to equalize the relative density levels in other regions of the protein (Supplementary Figure 11). C) Representation of EMMIVox ensembles of the E305-loop (residues 295–309) for one pentamer (decamer-orange and filament-blue) overlaid with ribbon structure of consensus model (tan). (D) Single filament and decamer turnover monomers overlaid and presented in blue and orange respectively presenting a hydrophobic stabilizing patch (I285, L300, and I309) and stabilizing salt-bridge (D239 and R298) in the ‘closed active’ loop state that is missing in the filament models. Distance measurements calculated across full EMMIVox ensembles represented as kernel density function between residues participating in ‘closed active’ loop state are presented demonstrating bias away from these contacts in the turnover filament form. (E) Same as (D) but representing the ‘tip’ of the loop showing E305 hydrogen bonding with S66 and H304 hydrogen bonding with N246 with quantification of these distances from the ensemble. In all cases, the filament oligomeric state under turnover conditions disfavored these interactions. (F) MD-refinement of the cryoEM filament turnover map revealed active site loop infiltration into the active site with R299 hydrogen bonding with active site residues E136 (top left) with distance quantification of R299 to E136 in the active site (top right). At bottom, overlay of all ten monomers per MD-refined model with loops in ribbon, ADP in stick, and the rest of the model is represented in surface. The filament loop is in an outward conformation from the active site at the base of helix 8.

To understand the structural basis underlying the *K*_*M, ammonia*_ defect, we inspected the interactions of active site residues and loops for differences across the oligomeric forms of GS. The GS active site forms a bifunnel shape with ATP gaining access to one subsite with glutamate and ammonia accessing the other subsite (Figure 3A). The glutamate/ammonia binding site is gated by a flexible loop that bears two key catalytic residues: R299, which interacts with glutamate substrate carboxyl group, and E305, which is predicted to deprotonate ammonium ions to ammonia, enabling the required nucleophilic attack of the y-glutamyl phosphate intermediate (Owji et al. 2022; Krajewski et al. 2008). Previously, crystal structures of human GS revealed a closed loop conformation in the presence of the transition state inhibitor methionine sulfoximine (PDB 2QC8). However, this loop exhibited weak electron density in the ADP-bound state (PDB 2OJW), which potentially indicates mobility of this element throughout the catalytic cycle.

Given the importance of this loop in gating glutamate and ammonia access to the active site, we examined how the E305-loop conformation and dynamics varied upon filament formation and substrate identity. All maps had similar resolution range (FSC_0.143_ between 1.95 – 2.27Å) and showed defined secondary structure and amino acid side chains in helix-12 (266–288) and beta sheet-11 (313–316) on either side of the loop at similar density contours (Supplementary Figure 11). The apo-filament cryoEM map showed well resolved density for the E305-loop with clear side chains, which support building an atomic model in a conformation we call the “closed-active state” (Figure 3B). Loop conformational heterogeneity was also influenced by nucleotide state, as the filament map with ATP only displayed partial density (Figure 3B). By contrast, the filament map collected during turnover displayed almost no density in the region, which is indicative of a greater degree of disorder. However, the E305-loop was partially restored in the cryoEM map of the turnover-decamer map (Figure 3B). This observation implicates the E305-loop conformational distribution as a potential factor contributing to the observed impaired kinetics of GS filaments. Taken together the E305-loop is sensitive to nucleotide state and also by filament state under turnover conditions.

The changes in density for the loop results from conformational heterogeneity that is ‘averaged out’ upon reconstruction. To provide an atomistic model of the conformational space explored by the E305-loop, we used Bayesian inference to balance experimental cryoEM data with accurate physico-chemical molecular dynamics as implemented in EMMIVox ensemble refinement (Hoff et al. 2024). Across different conditions, the conformation of the loop was dominated by the closed-active loop conformation (Figure 3C). However, the turnover-filament model displays distended loop conformations diverged from the active-closed state that we turn collectively as the ‘open ensemble’ of loop conformations (Figure 3C). The E305-loop (residues 299 – 311) sampled a more diverse ensemble (with root mean square fluctuation values of greater than 10Å) in the turnover filament state, with the turnover decamer and apo-filament displaying reduced variability (Supplementary Figure 12). By contrast, across all states, the remainder of the active site maintained similar fluctuations (Supplementary Figure 12).

We next examined the principal contacts that serve to stabilize the ‘active closed’ state of the loop (Figure 3D,E). We observe multiple residue contacts that appear stabilizing including a hydrophobic pocket formed by L300, I285, and I309, a salt-bridge formed between R298 and D239, and a hydrogen bond between H304 and N249 (Figure 3D,E). All these interactions displayed favored distances in the decamer-turnover compared to filament turnover ensembles. A major consequence of this increased loop mobility is that Arg299 can “invade” the active site (Figure 3F). We quantified the distance distribution between R299 and E136 in our turnover-decamer and turnover-filament ensembles and found a substantial bias towards R299 infiltration of the active site in the filament ensemble that is absent in the turnover decamer ensemble (Figure 3F). The conformation of Arg299 could potentially sterically hinder substrate access to the active site and thus contribute synergistically with loop mobility to the observed *K*_*M, ammonia*_ defect. Collectively, these results suggest that biasing of conformational distribution of the loop is important for tuning activity in GS.

### Global conformational change coordinates E305-loop dynamics

**Figure 4.**
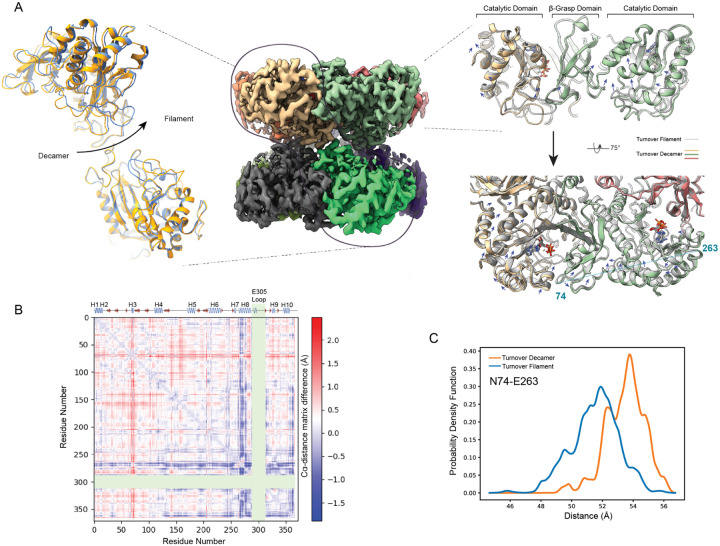
Global conformational change is associated with loop conformational heterogeneity under turnover conditions. (A) Left: rotational motion about the pentameric interface observed where models were aligned on the bottom monomer and rotation seen in the upper monomer is noted (decamer in yellow and filament in blue). Middle: representation of the decameric turnover cryoEM map colored per monomer with outlines showing relevant areas shown at left and right. Right: monomeric interface within a pentamer overlay of the turnover filament model (gray) with turnover decamer model colored by monomer (tan, green, and red). Ligands are colored by atom type. Arrows indicate conformational change from filament to decamer state. The largest Ca distance change not including the E305-loop is indicated between residues 74 and 263 from across the pentamer. (B) Ca distance difference matrix for chain G between turnover decamer and turnover filament models with the E305-loop (residues 289–312) and the far C-terminal peptide (369–373) omitted with a cartoon secondary structure representation of a monomer on top and distance color legend at right demonstrating that the tip of the B-grasp region (60–76) and helix 8 (residues 262–282) showing the largest conformational differences. (C) Ca distance differences for residues 74 and 263 between the turnover filament and turnover decamer ensembles demonstrating a robust 2 angstrom distance difference.

We next asked what conformations or structural features underlie these changes to E305-loop conformational space and how filament formation promotes an ‘open ensemble’. Utilizing our EMMIVox refined models as a starting point, we assessed global conformational changes between the turnover-filament and turnover-decamer structures that might influence the local conformation of the loop. First, we found a rotational conformational change (1.2°) apparent about the pentamer-pentamer interface (Figure 4A). Given that this conformational change could impact the local conformation at each composite active site by altering monomeric interfaces we explored how this global conformational change related to the overall conformation of each monomer. We observed a ‘crimping’ conformational change per monomer where in the filament structure the β-grasp domain and the catalytic domain are rotated towards each other and away from their respective active sites (Figure 4A).

We next sought to define the regions within each monomer most altered upon filament formation by performing a Cα-distance matrix subtraction analysis (Figure 4B; Supplementary Figure 13). As expected, the E305-loop conformational heterogeneity dominated this analysis, however, we also observed an ~2Å shift in the tip of the β-grasp domain (residues 70–76) and helix-8 which abuts the active site and immediately precedes the E305-loop (Figure 4B; Supplementary Figure 13). We found the largest Cα-distance change between these two regions, specifically, between N74 and E263 displaying a distance change of 2.05Å (Figure 4A). Finally, to understand how prevalent this global conformational change is we quantified this Cα-distance in the full turnover-filament and turnover-decamer ensembles and found persistent distance distribution changes of ~2Å between these two ensembles (Figure 4C). Thus, we propose that this global conformational change serves to influence E305-loop conformational distribution via two primary mechanisms via helix 8 repositioning away from the active site in the filament form which: 1) hinders E305-loop ability to completely close over the ammonia binding site (residues S66, S67, and D76;(Figure 4B,C) and 2) disfavors E305-loop contacting closed-state stabilizing residues (Figure 3D,E).

### E305-loop conformational space is crucial for Michaelis-complex formation

Since all ensembles, including the apo-filament, could sample both the ‘active closed’ and ‘open-ensemble’ states, we next pursued mutagenesis to more directly test the importance of the loop flexibility on catalysis. We created alanine mutants of residues D239, R298, L300, I309, H304, and E305. Each of these residues reside on the E305-loop with the exception of D239 which participates in a salt-bridge interaction with R298 on the loop. E305 was chosen to be an active site control, which does not appear to stabilize the closed state of the loop, but rather, the closed state of the loop positions E305 near the proposed ammonia binding site (Moreira, Ramos, and Fernandes 2017; Issoglio et al. 2016; Frieg et al. 2016).

**Figure 5:**
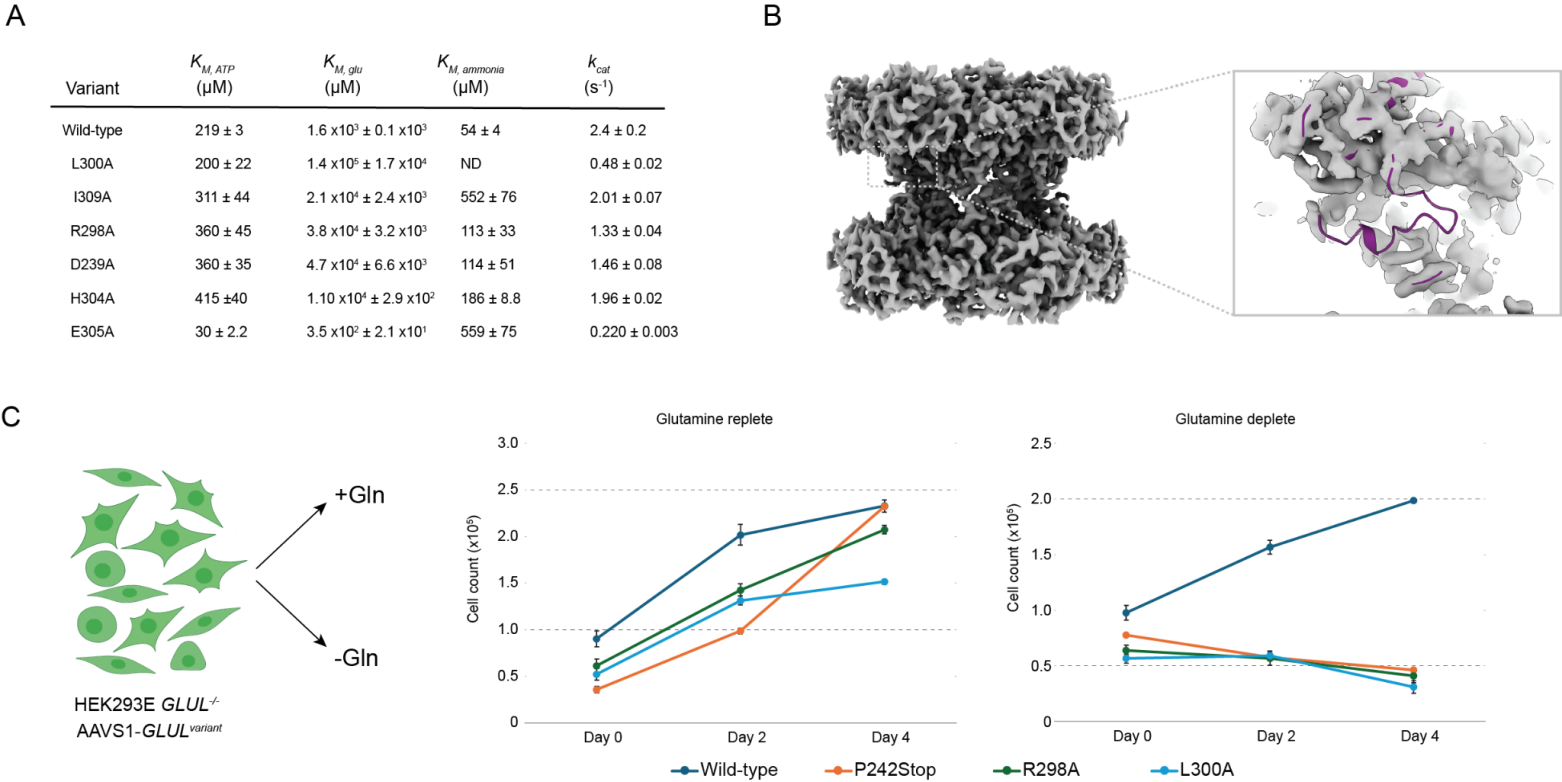
Glutamine synthetase E305-loop conformation controls Michaelis-complex formation. A) Steady-state kinetic parameters for wild-type GS (also displayed in Figure 2) and six point-mutants of GS, L300A, I309A, R298A, D239A, H304A, E305A as assessed by the coupled-ADP release assay (methods). (B) cryoEM density map for R298A decamer under turnover conditions displayed in full (left) with inset focused on loop density (right) demonstrating complete loss of the E305-loop for this variant. (C) Schematic of *HEK293E GLUL−/−* cell line auxotrophic for glutamine is reconstituted with single genomic integration of individual *GLUL* variants (methods) and used to assess glutamine prototrophy. All variant cell lines grow equivalently in glutamine replete media conditions (middle) but only the wild-type *GLUL* variant could support cell growth and survival under glutamine deplete conditions, whereas R298A, L300A, and a premature stop codon at P242 all led to cell death.

We measured the steady-state Michaelis-Menten parameters of all variants (Figure 5A). The E305A variant principally impacted *K*_*M, ammonia*_ (~10 fold) as would be expected from this residue’s predicted role of deprotonating ammonium ions in preparation for nucleophilic attack. The E305A variant was also the only variant to display a large *k*_*cat*_ effect (~10 fold) which is also expected given its presumed role in priming ammonium to ammonia in the active site (Moreira, Ramos, and Fernandes 2017; Issoglio et al. 2016; Frieg et al. 2016). The *K*_*M, ammonia*_ parameter was also impacted for all other loop variants tested by 2–10 fold. However, the principal kinetic impact for other loop variants was on the *K*_*M, glutamate*_. The magnitude of *K*_*M, glutamate*_ change ranged from 100 fold in L300A to 5 fold in H304A. Disruption of the hydrophobic interface of L300, I309, and I285 appeared to have largest impact on *K*_*M, glutamate*_, followed by disruption of the R298*D239 salt bridge (50-fold change for both variants), followed lastly by disruption of the H304*N249 hydrogen bond.

The *K*_*M, ammonia*_ was impacted but generally less so than *K*_*M, glutamate*_ in E305-loop variants. This result likely due to the use of saturating glutamate concentrations required to estimate *K*_*M, ammonia*_ where the majority of active sites are glutamate bound, or y-glutamyl phosphate formed, which will have had already promoted the closed-active loop state via amino acid carboxylate-R299 interaction, thus restricting the potential scope of loop conformational variability. Even with such a presumed stabilization, the full Michaelis-complex formation is still impacted for the final ammonia substrate binding which is likely a consequence of the biased conformational state of the E305-loop. Taken together, these data indicate that mutations outside the active site to the E305-loop stabilizing interactions have large impacts to the *K*_*M*_ for substrates that bind to half-active sites gated by the loop. These mutations do not have pronounced effects to the overall *k*_*cat*_ however, indicating that while Michaelis-complex formation is hindered, the rate-limiting step of catalysis is unimpacted.

To test the model that loop biasing impacts *K*_*M*_ values for GS, we structurally characterized the decameric form of loop variant R298A under turnover conditions. The final reconstruction yielded an overall map of FSC_0.143_ of 1.95 Å with high local resolution values across the map (Supplementary Figure 14). Importantly, this map, despite being the highest global resolution map reconstructed, did not display any appreciable E305-loop density (Figure 5A), similar to the turnover filament (Figure 3B). Thus, the R298A mutant, even in the decameric form, would rarely adopt the active-closed conformation, explaining the *K*_*M, glutamate*_ and *K*_*M, ammonia*_ defects resulting from this mutation. Collectively, these data suggest a connection between loop ensemble and catalytic activity for human GS that is principally manifested in *K*_*M*_, changes for amino acid and ammonium substrates.

### Loop variants are unable to support glutamine auxotrophy

Since our data suggested that altering loop dynamics via mutation or filamentation could alter *K*_*M*_, we next wanted to test the effect of perturbing the loop on the overall cellular activity of GS. Most human cells require exogenous glutamine to support growth in tissue culture unless they express GS. Indeed, GS expression has been used as an auxotrophic marker for antibody production HEK293E GLUL−/− cell line (Yu, Lee, and Lee 2018). Starting from this auxotrophic HEK293E GLUL −/− cell line, we used CRISPR to introduce an attP landing pad (Matreyek, Stephany, and Fowler 2017; Matreyek et al. 2020), to facilitate stable cell line generation expressing individual GS variants. To create each mutant line, we transfected with Bxb1 and cognate attB GLUL variant DNA. GS variants were then overexpressed from a pTet promoter driven by doxycycline and tested for growth in the presence and absence of glutamine supplementation (Figure 5C)

Glutamine prototrophy of this cell line was confirmed as high cell proliferation was observed for wild-type GS in glutamine deplete media while there was a lack of survival for a variant bearing a premature stop codon inserted at P242 (Figure 5C). We assessed glutamine auxotrophy for GS filament interface variants C53A and K52A (Supplementary Figure 15). As expected, in the HEK293 model, both variants supported glutamine auxotrophy to similar levels as wild-type. This system is not likely to be sensitive to the feedback inhibition mediated by filamentation to reduce glutamine production as ammonia levels in HEK293 cells typically exceed those levels seen in healthy tissues by up to 100-fold (Rajendra et al. 2011). However, the two loop mutants, R298A and L300A, could not support growth under glutamine-deplete conditions displaying the same phenotype as P242stop. The *K*_*M, glutamate*_ for R298A and L300A are outside the normal steady state concentrations of intracellular glutamate, suggesting that loop conformational control of the Michaelis-complex can occur in the cell to directly impact metabolic flux through GS.

## Discussion

**Figure 6:**
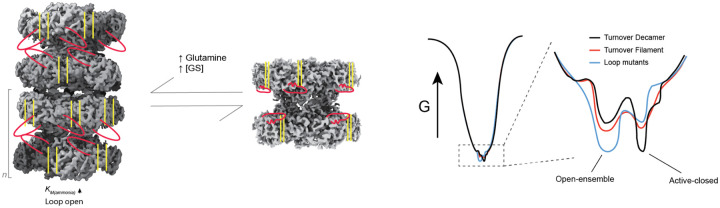
Filament formation tunes E305-loop dynamics to modulate enzyme activity. Left: cryoEM map of the turnover filament which includes a higher *K*_*M, ammonia*_ and open E305-loop indicated at bottom and by an ‘open’ red colored E305-loop cartoon. Also indicated is the global conformational change that opens up the monomeric interfaces about the active site (yellow lines). This form of the enzyme is increased with increased GS concentration and/or glutamine concentration and is different from the decamer which includes a more closed active site and less conformationally heterogeneous E305-loop. Right: proposed conformational landscape of GS wherein the global fold of the enzyme is largely retained and subtle conformational/ensemble changes found in the low thermodynamic basin are present. Inset: the E305-loop ensemble is depicted for the turnover decamer, turnover filament, and E305-loop mutants wherein the decamer slightly favors an active-closed state, the filament an open-ensemble, and loop variants significantly favor the open-ensemble state.

We propose a model for glutamine synthetase regulation through filament formation that alters the conformational landscape of the active site gating E305-loop (Figure 6). We show that filament formation is driven both by higher enzyme concentrations and glutamine binding at the filament interface. Importantly, the protein concentrations used in our cryoEM experiments are within the range of intracellular GS concentrations in S. cerevisiae (Engel et al. 2025) and human cell lines (Wiśniewski et al. 2014). Additionally, we found that physiological concentrations of glutamine can stabilize filamentous GS structures.

GS is an ancient enzyme that is found in all domains of life as it serves a critical crossroads between carbon and nitrogen metabolism. Given its centrality to numerous metabolic pathways, GS is subject to sophisticated intracellular regulation across the domains of life. E305-loop flexibility has been noted in x-ray crystallographic structures of human GS (Krajewski 2008) and in other homologs too (Tecson et al. 2025) supporting a model of functional flexibility of this structural element. The most well studied is type I-beta GS (GSIB) found in gram negative bacteria which contains a ‘regulatory loop’ that abuts the E305-loop at the active site. The regulatory loop can be covalently adenylated by a bifunctional enzyme that is sensitive to glutamine levels and adenylation inhibits GS (Eisenberg et al. 2000). Given our demonstration of the importance of maintaining optimal E305-loop flexibility in human GS, it is perhaps unsurprising that a large post-translational modification applied to a regulatory loop of GSIB could have a dramatic functional impact. The E305-loop thus is likely a hotspot for GS regulation via multiple distinct routes across evolution.

Here, we observe a connection between the filamentation and E305-loop flexibility. There is evidence that GS homologs from bacteria, archaea, and eukaryotes also form a filamentous, head-on-head structure, but their structural and functional consequences are incompletely understood. In E. coli, GS filaments could be formed from supraphysiological levels of select divalent cations that are chelated by adjacent helices across repeating dodecamer units. While high structural resolution was achieved, the catalytic and cellular consequences of filament formation were not explored. Furthermore, E. coli filaments dissolved under turnover conditions leaving the functional relevance unclear. By contrast, in S. cerevisiae, GS filaments were demonstrated to negatively regulate GS activity in the cell and shown to be a crucial adaptation upon starvation conditions (Petrovska et al. 2014). Here, we provide a structural rationale describing how filament formation, stabilized by product, can structurally alter GS E305-loop to tune the substrate-limiting Michaelis constant for ammonia. Thus, the glutamine stabilized filament formation model for human GS represents a new mode of product-based allosteric feedback inhibition for this enzyme class.

Human GS is expressed in many tissues to serve different metabolic needs. Translation and subsequent degradation of GS can take multiple hours to achieve robust expression changes (Arad, Freikopf, and Kulka 1976; T. Van Nguyen et al. 2016; Thang Van Nguyen et al. 2017; Thang Van Nguyen 2021; Zhao et al. 2025) which is potentially insufficient to meet the changing metabolic needs of a given cell. Therefore, regulated enzyme activity through filament formation represents a mode of high temporal control over enzyme activity as it is not reliant on transcription, translation, or degradation of enzymes. Additionally, the Michaelis constant for ammonia report herein (57 μM for decameric GS and 170 μM for filamentous GS) corresponds closely to the concentrations of ammonia measured in cells, which suggests that this modulation of GS activity could have an immediate impact on intracellular turnover rates.

Moreover, our observations support the concept that allostery operates through ensemble redistribution rather than through discrete conformational switches. The filament state of human GS does not simply toggle the E305-loop between rigidly defined “open” and “closed” states, but rather shifts the probability distribution across a spectrum of conformations that collectively impact enzyme activity. This is similar to observations in IMPDH2 and PRPS filaments (Hvorecny et al. 2023; M. C. Johnson and Kollman 2020), where assembly modulates the conformational landscape accessible to regulatory elements. Both loop dynamics and filamentation are frequently implicated in tuning activity of enzymes; here a product feedback mechanism links these two concepts together. Collectively, the filament formation of human GS described here is a complex manifestation of ensemble-based allostery to enable timely regulation of enzyme activity.

## Methods

### Expression and purification of scarless human glutamine synthetase

An expression vector for a 6xHIS-conjugated human glutamine synthetase was ordered from ATUM. The vector was transformed into BL21(DE3) *E. coli* cells and grown overnight at 37 °C in Luria broth with 100 μg ml−1 carbenicillin. 5 mL of this starter culture was added to 1 L of the same medium and allowed to grow at 37 °C until an OD600 of 0.6–0.8 was reached. At this point, expression of GS was induced with 0.5 mM IPTG and the temperature was reduced to 18 °C for 16 – 18 hours. The cells were harvested by centrifugation at 4500 rpm for 15 minutes at 4 °C. The cell pellets were transferred to a 50-mL conical tube and stored at −80 °C.

For purification, cells were thawed and resuspended in 60 mM HEPES pH 7.6, 50 mM NaCl, 50 mM MgSO_4_, 0.5 mM TCEP, 10% glycerol followed by lysis by sonication. Lysate was clarified by centrifugation and loaded onto HisTrap FF 5 mL (Cytiva) columns using a peristaltic pump, washed with 60 mM HEPES pH 7.6, 50 mM NaCl, 50 mM MgSO_4_, 0.5 mM TCEP, 10% glycerol, 25 mM imidazole, and eluted with 60 mM HEPES pH 7.6, 50 mM NaCl, 50 mM MgSO_4_, 0.5 mM TCEP, 10% glycerol, 250 mM imidazole. Eluate was concentrated before being aliquoted and flash frozen and stored at −80°C. Thawed aliquots were loaded onto a Superose 6 10/300 column preequilibrated with 60 mM HEPES pH 7.6, 50 mM NaCl, 50 mM MgSO_4_, 0.5 mM TCEP and assayed for activity immediately after size-exclusion chromatography.

### Expression and purification of scarless human glutamine synthetase

Scarless human glutamine synthetase was produced by either of two independent methods: N-terminal His-SUMO tagging with Ulp1 cleavage or C-terminal intein-chitin binding domain-His with DTT induced self cleavage. Both purification methods yielded highly functional GS, however, better yields were obtained via the His-SUMO tagging approach.

Human glutamine synthetase was codon optimized for E. coli (ATUM biosciences) and appended with a C-terminal Mxe GyrA intein domain (NEB IMPACT) followed by a chitin-binding domain (CBD) from *Bacillus circulans* (NEB IMPACT) cloned in-frame with a 3′ His_6_-tag and transformed into BL21(DE3) (NEB). Two liters of Terrific Broth (Fisher) were pre-warmed to 37 °C before being inoculated with 10 ml overnight starter culture. Cultures were grown to 0.6 < OD600 < 1.0 and induced with 0.5 mM isopropyl β-D-1-thiogalactopyranoside (GoldBio) and protein expression was allowed to continue for 3 hours. Cells were pelleted at 3,500 r.c.f. for 20 min at 4 °C, resuspended in base buffer (60 mM HEPES pH 7.6, 50 mM NaCl, 50 mM KCl, 10 mM MgCl_2_, 0.5 mM TCEP) supplemented with 2 mg/mL lysozyme, benzonase, and cOmplete^™^, EDTA-free Protease Inhibitor Cocktail (Roche) and stored at −80°C until purification.

For the purification of GS-intein-CBD-His, cells were thawed and lysed by sonication. Lysate was clarified by centrifugation and loaded onto HisTrap FF 5 mL (Cytiva) columns using a peristaltic pump, washed with base NiA buffer (60 mM HEPES pH 7.6, 50 mM NaCl, 50 mM KCl, 10 mM MgCl2, 25 mM imidazole), and eluted with base NiB buffer (60 mM HEPES pH 7.6, 50 mM NaCl, 50 mM KCl, 10 mM MgCl2, 250 mM imidazole). Eluates were spiked with DTT to a final concentration 100 mM and stored overnight at 4C to allow for intein self-cleavage. Cleaved GS was buffer exchanged into NiA buffer via application to a HiTrap 26/10 Desalting column (Cytiva). His_6_-tagged Mxe GyrA intein-CBD was removed through an ortho NiNTA where sample was applied to a HisTrap High Performance 5 mL column (Cytiva) and the flow through fraction was collected, concentrated in an Amicon 30k MWCO centrifugal filter, spiked with glycerol to a final concentration of 10%, aliquoted, and flash frozen for long term storage at −80°C.

Human glutamine synthetase was codon optimized for E. coli (ATUM biosciences) and subcloned into a pET29a vector bearing an inframe 5′ His_6_-SUMO tag and transformed into BL21(DE3). Two liters of Terrific Broth (Fisher) were pre-warmed to 37 °C before being inoculated with 10 ml overnight starter culture. Cultures were grown to 0.6 < OD600 < 1.0 and induced with 0.5 mM isopropyl β-D-1-thiogalactopyranoside (GoldBio) and protein expression was allowed to continue for 3 hours. Cells were pelleted at 3,500 r.c.f. for 20 min at 4 °C, resuspended in base buffer (60 mM HEPES pH 7.6, 50 mM NaCl, 50 mM KCl, 10 mM MgCl_2_, 0.5 mM TCEP) supplemented with 2 mg/mL lysozyme, benzonase, and cOmplete^™^, EDTA-free Protease Inhibitor Cocktail (Roche) and stored at −80°C until purification.

For the purification of His_6_-SUMO-GS, cells were thawed and lysed by sonication. Lysate was clarified by centrifugation and loaded onto HisTrap FF 5 mL (Cytiva) columns using an AKTA start, washed with base NiA buffer (60 mM HEPES pH 7.6, 50 mM NaCl, 50 mM KCl, 10 mM MgCl2, 25 mM imidazole; 250 mL), and eluted with base NiB buffer (60 mM HEPES pH 7.6, 50 mM NaCl, 50 mM KCl, 10 mM MgCl2, 250 mM imidazole; 50 mL).

Eluates were spiked with Ulp1 (see purification below) and dialyzed overnight at 4C against base buffer. Cleaved GS was separated from His_6_-tagged His-SUMO through an ortho NiNTA where sample was applied to a HisTrap High Performance 5 mL column (Cytiva) and where scarless GS could elute with application of base buffer with 25 mM imidazole. Scarless GS was concentrated in an Amicon 30k MWCO centrifugal filter, spiked with glycerol to a final concentration of 10%, aliquoted, and flash frozen for long term storage at −80°C.

Prior to experimentation, a scarless GS aliquot was thawed, filtered through a 0.22 μm filter, and applied to a Superose 6 increase 10/300 size-exclusion column pre-equilibrated with base buffer. Unless otherwise stated, the decameric peak corresponding to a 16 mL retention volume was collected and stored at 4C throughout the duration of experimentation. Activity of this peak was unchanged for up to 2 weeks and all biochemical experiments were performed immediately upon purification.

### Purification of Ulp1 Sumo protease

The expression vector encoding His_6_-Ulp1 protease was a gift from the Verba lab at UCSF obtained originally from Addgene 64697. Ulp1 was expressed and purified as previously described (Guerrero, Ciragan, and Iwaï 2015) with minor adaptations detailed below. Briefly, 2L of Terrific broth were pre warmed to 37°C before inoculation with 10 mL of a starter culture of BL21(DE3) cells bearing the His_6_-Ulp1 expression vector. Cells were grown to 0.6 < OD_600_ < 0.8 before temperature was dropped to 20°C and expression was induced with 0.5 mM IPTG overnight. Cells were pelleted at 3,500 r.c.f. for 15 min at 4°C and stored in a 50 mL tube at −80°C until preparation.

Cells were resuspended in Lysis buffer (50 mM HEPES pH 8, 350 mM NaCl, 0.5 mM TCEP, 10 mM imidazole, 2 mg/mL lysozyme, benzonase, and cOmplete^™^ EDTA-free Protease Inhibitor Cocktail (Roche). Lysate was clarified by centrifugation and loaded onto HisTrap FF 5 mL (Cytiva) columns using an AKTA start, washed with 25 mL of Lysis buffer followed by 25 mL of Buffer A (50 mM HEPES pH 8, 350 mM NaCl, 0.5 mM TCEP, 10 mM imidazole) and 25mL of Buffer B (50 mM HEPES pH 8, 350 mM NaCl, 0.5 mM TCEP, 30 mM imidazole). Finally Ulp1 was eluted with 25 mL of Buffer C (50 mM HEPES pH 8, 200 mM NaCl, 0.5 mM TCEP, 300 mM imidazole). Pure fractions were combined and dialyzed against 50 mM HEPES pH 8, 200 mM NaCl, 10 mM EDTA, 0.5 mM TCEP, 20% glycerol). Ulp1 was concentrated to 2 mg/mL and flash frozen in 250 uL aliquots and stored at −80°C.

### Negative stain microscopy

GS was prepared to ~0.01 – 0.05 mg/mL in base buffer prior to application to glow-discharged carbon-coated grids, stained with 0.7% uranyl formate and imaged on a T12 (FEI) operating at 120 kV. Images were acquired at ×57,000 magnification on a Gatan Rio camera. Two-dimensional classification and averaging was conducted using cisTEM.

### BSG and BMOE crosslinking of GS filament interface

BMOE (bismaleimidoethane; ThermoFisher) and BSG (bis(sulfosuccinimidyl) glutarate-d0; ThermoFisher) powder was thawed to room temperature and reconstituted in DMF to a concentration of 50 mM, aliquoted, flash frozen in liquid nitrogen, and stored at −80°C. Protein samples were diluted to concentrations noted in base buffer, reacted with crosslinker to a final concentration of 0.5 mM for 10 mins at room temperature followed by quench in SDS-PAGE sample buffer supplemented with 100 mM of either NH_4_Cl (to quench BSG) or DTT (to quench BMOE). Protein concentrations were normalized after quench prior to SDS-PAGE analysis.

### Michaelis-Menten analysis using coupled ATPase assay

ATP-hydrolysis rates were determined using an NADH-coupled assay (pyruvate kinase and lactate dehydrogenase; Sigma) as described previously. Briefly, GS was diluted into ATPase mix (final concentrations: 1 mM NADH, 7.5 mM phosphoenolpyruvate, 6 U/mL pyruvate kinase, and 6 U/mL lactate dehydrogenase), applied to a 384-clear bottom plate (Corning) in 10 uL final volume. Steady-state depletion of NADH was assessed by measuring the absorbance at 340 nm in a Spark 10M multimode plate reader (Tecan). Single substrate concentrations were varied under saturating conditions of other substrates as determined by primarily kinetic assessment. Solution pathlength was manually determined per experiment through titration of NADH and used to calculate ATPase rate. Resultant enzyme normalized velocities were plotted against variable substrate concentration and fit using Equation 1:

Eqn. 1 $$v_{o}=\frac{{k_{cat}}^{*}[\text{substrate}]}{K_{M}+[\text{substrate}]}$$

The low concentrations of ammonium required to assess *K*_*M, ammonia*_ (< 10 μM) resulted in small changes in total NADH concentration (<1%) relatively quickly and therefore use of full progress curve analysis was pursued as described(K. A. Johnson 2019), which captures the rate of substrate depletion or production formation over time as [S] decreases by fitting to the differential equations inherent to the Michaelis-Menten kinetic model. Here, saturating glutamate and ATP concentrations were used in combination with three starting ammonia concentrations (750 μM, 375 μM, and 188 μM) and the NADH-coupled assay components as above in 2X concentration before being mixed with 2X enzyme (0.1 mg/mL final) and the resultant 10 uL reactions (n = 8) were monitored for 5 mins at room temperature in a Spark 10M multimode plate reader (Tecan). Replicates were averaged and the average standard deviation per time point was imported in Kintek Explorer. A standard Michaelis-Menten model was applied to the simulation defined by *k*_*1*_, *k*_*-1*_, *k*_*2*_, *k*_*-2*_, and *k*_*3*_ as defined in Equation 4.


Scheme 1. $$E+S\underset{k_{-1}}{\overset{k_{l}}{⇄}}ES\underset{k_{-2}}{\overset{k_{2}}{⇄}}EP\overset{k_{3}}{\to }E+P$$

Eqn. 2 $$k_{cat}=\frac{k_{2}k_{3}}{k_{2}+k_{-2}+k_{3}}$$

Eqn. 3 $$K_{M}=\frac{\left(k_{2}+k_{-1}\right)\left(k_{-2}+k_{3}\right)}{k_{1}\left(k_{2}+k_{-2}+k_{3}\right)}$$

Eqn. 4 $$\frac{k_{cat}}{K_{M}}=k_{1}\frac{k_{2}k_{3}}{k_{2}k_{3}+k_{-1}\left(k_{-2}+k_{3}\right)}$$

Derived elementary step parameters and their associated uncertainties were used to determine the steady state kinetic parameters with error propagated through each step. The derived *k*_*cat*_ parameters from this procedure were in agreement with those derived under the traditional initial rate procedure.

### Sample preparation for cryoEM analysis

Frozen, mostly pure, GS aliquots were thawed and further purified by size-exclusion chromatography on a superose 6 10/300 column where fractions corresponding to the decamer oligomeric state (~16 mL) were collected, quantified by A_280_ and tested for activity. Grids (Quantifoil 1.2/1.3 Cu 300 (with or without +2nm C) or Quantifoil 2/2 Au 200) were glow discharged in a Pelco-Easi Glo (15 mA, 10–15s on, 10–15s off) before sample application. Enzyme (0.3–1.3 mg/mL final) in apo (buffer added) or turnover (5 mM ATP, 25 mM ammonium chloride, and 50 mM glutamate; reacted for 53–600s and within steady-state kinetic regime except for the turnover-filament dataset where near full conversion of substrate to product was expected) or ATP bound (5 mM ATP) at room temperature before application to grid held at 4° or 18°C in a Mark IV Vitrobot (ThermoFisher) prior to vitrification. For turnover conditions, all data were collected on Quantifoil 1.2/1.3 Cu 300 +2nm C grids where reactions were initiated at room temperature immediately before being applied to grid in a Vitrobot Mark IV (Thermo) pre-equilibrated to 18°C with 100% humidity and allowed to adsorb to ultrathin carbon layer for 30s before blotting (blot force 3, 11s, Whatman Filter Paper #1) and plunge frozen into liquid ethane. For apo- and ATP-filament datasets, grids were held at 4°C with 100% humidity, blotted 1–3 times with sample, before plunge frozen in liquid ethane. Observation of short filamentous structures was observed with and without depositing 2nm continuous carbon film on grid and preparations

### CryoEM data collection

Wild-type decamer turnover, time-resolved datasets, and R298A decamer turnover GS sample grids were loaded onto a Titan Krios G3i 300 keV microscope (TEM Gamma; SLAC S2C2 Facility, Menlo Park, CA) with a BioQuantum energy filter (20 kV) and Gatan K3 Ceta-D direct detector operating at a nominal magnification of 105,000x corresponding to a physical pixel size of 0.86 Å. A zero-loss peak optimization and gain reference was taken ahead of each data collection. Each dataset was set to a total accumulated dose rate of 40 e-/Å^2^ per micrograph. Each movie was split into 75 frames and a nominal defocus range was set between −0.8 to −2.0 μm. Fringe-free illumination was utilized and image shift was used to collect multiple images within a single hole and beam tilt was adjusted to achieve coma-free alignment during image shift. Automated data acquisition was performed with EPU software.

The low resolution turnover filament dataset was collected at SLAC S2C2 Facility (Menlo Park, CA) on a Talos Arctica G2 200 keV microscope with a Gatan K3 direct detector operating at a nominal magnification of 106,000X corresponding to a physical pixel size of 0.86 Å. A zero-loss peak optimization and gain reference was taken ahead of each data collection. The dataset was set to a total accumulated dose rate of 40 e-/Å^2^ per micrograph. Each movie was split into 40 frames and a nominal defocus range was set between −0.8 to −2.0 μm. Automated data acquisition was performed with EPU software.

The turnover filament dataset was collected at the UCSF CryoEM Core facility where grids were loaded onto a Titan Krios G2 300 keV microscope (Krios 1) with a 20kV energy filter and Gatan K3 direct detector operating at a nominal magnification of 106,000x corresponding to a physical pixel size of 0.82 Å. A zero-loss peak optimization and gain reference was taken by facility management each week. Total accumulated dose rate of 45 e-/Å^2^ per micrograph. Each movie was split into 75 frames and a nominal defocus range was set between −0.8 to −2.0 μm. Fringe-free illumination was utilized and image shift was used to collect multiple images within a single hole and beam tilt was adjusted to achieve coma-free alignment during image shift. Automated data acquisition was performed with SerialEM software.

Apo- and ATP-Filament datasets were collected at the Arnold & Mabel Beckman center for cryo-electron microscopy at the University of Washington where grids were loaded onto a Titan Krios G3 300 keV microscope (Krios 1) with a 20kV Gatan BioQuantum energy filter and Gatan K3 direct detector operating at a nominal magnification of 105,000x corresponding to a physical pixel size of 0.843 Å. Total accumulated dose rate between 60 and 64.2 e-/Å^2^ per micrograph.

### CryoEM image processing and model building

Movies were aligned, motion-corrected, and dose-weighted using Patch Motion Correction in cryoSPARC (v3.1 and above) or cryoSPARC Live, except for the turnover filament dataset which was motion-corrected and dose-weighted on-the-fly with MotionCor2 (v1.3.0). Contrast transfer function (CTF) estimation was performed in cryoSPARC using the Patch CTF except for the turnover filament dataset which was estimated using CTFFIND4 ‘on-the-fly’ as part of the Scipion (v2.0) software suite. Micrographs with poor CTF fit (generally >5 Å and/or containing crystalline ice) were discarded. All further processing steps were performed in cryoSPARC (v3.1 or higher) where blob picked particles were classified in two dimensions and non-background particles were subject to either further rounds of template picking and 2D classification or used for 3D homogeneous refinement using either *ab initio* prepared input maps or using maps from other datasets as 3D refinement input. Where applicable, particles were further selected with either or a combination of heterogeneous refinement and/or 3D classification as shown in the supplemental figures. Homogeneous or non-uniform refinement was utilized for final refinement and reconstructions and typically included a symmetry operator (D5 for filament reconstructions, C5 for R298A decamer turnover reconstruction and C1 for wild-type decamer turnover reconstruction). Local map resolution was estimated in cryoSPARC for the final maps.

An initial model, PDB 2OJW, was used to build the apo-filament model and the apo-filament model was used as the initial model for all subsequent model building. Models were built iteratively using the ISOLDE (v1.7) plugin for ChimeraX (v.1.7 and above), Coot (v0.9.8.7), and Phenix (v1.20.1; Real Space Refinement and CryoEM Comprehensive Validation). Models were additionally validated with Q-score as a plugin function for ChimeraX (Supplementary Table 1).

### MD-based cryoEM ensemble refinement

Initial models of glutamine synthetase under turnover conditions in the filament (complexed with ATP and MG) and decamer form (complexed with ADP, ATP and MG), as well as a model of glutamine synthetase in the apo-filament form generated from cryoEM data were processed using the CHARMM-GUI server to initialize molecular dynamics simulations (Jo et al. 2008). Each system was solvated in a triclinic box such that the edge of the box was 1.0 nm away from the closest atom of each glutamine synthetase model. K+ and Cl- salts were added to the system at a concentration of 0.15 M, along with additional ions to neutralize the charge of each system. The CHARMM36m forcefield was used for the proteins and the CHARMM General Forcefield (CGenFF) (Vanommeslaeghe et al. 2010) was used for the ATP and ADP molecules present in the turnover condition systems. Water molecules were modeled using the mTIP3P model (MacKerell et al. 1998). All simulations were carried out using a leap-frog algorithm with a 2 fs timestep. The smooth particle mesh Ewald method (Essmann et al. 1995) was used for calculating electrostatic interactions within a cutoff distance of 1.2 nm. Van der Waals interactions were gently decreased at 1.0 nm and cutoff at 1.2 nm. All simulations were performed using GROMACS v 2021.5 (Abraham et al. 2015) paired PLUMED v 2.9 (Tribello et al. 2014).

#### Details of EMMIVox Simulations.

Refined single-structure models and structural ensembles of the GS in the apo-filament, turnover filament, and turnover decamer forms were generated using EMMIVox (Hoff et al. 2024). In brief, EMMIVox is a Bayesian inference approach to determine single-structure models and structural ensembles from 3D cryoEM maps. The direct utilization of experimental cryoEM density and 3D half-maps in the Bayesian framework of EMMIVox automatically balances experimental information with accurate physico-chemical models. Both single structure refinement and ensemble modelling followed the standard EMMIVox protocol (Hoff et al. 2024). CryoEM maps for each GS system and their associated half-maps were pre-processed prior to EMMIVox simulations. Correlated voxels within the experimental 3D maps were removed. This was achieved by first selecting all voxels within 3.5 Å of the model and removing correlated data points with a correlation greater than a pearson coefficient threshold of 0.8. Next, the difference between voxel density for the two half-maps of each system were used to generate a lower bound for the error parameter associated with each density voxel in the EMMIVox framework. Equilibration and production simulations were performed as follows. First, each system was energy minimized using the steepest gradient descent approach. Next, a 10 ns long NPT equilibration was performed using the Bussi-Donadio-Parrinello thermostat (Bussi, Donadio, and Parrinello 2007) and Berendsen barostat (Berendsen et al. 1984) set at 300K and 1 atm respectively. Next, 2 ns long NVT simulations were formed using the same thermostat at 300K. Positional restraints were applied to the NPT and NVT equilibration simulations. Next, an optimal scaling factor between the predicted and observed cryoEM maps was determined using the NVT equilibration simulation trajectory and the PLUMED *driver* tool. Scaling factor values between 0.5 and 1.8 were scanned at 0.05 intervals, with the scaling factor resulting in the lowest EMMIVox hybrid energy score selected for use in the production simulations. B-factors for each chain in the GS were optimized independently without the use of symmetry constraints.

Single-structure refinement production simulations were performed in the NVT ensemble using the thermostat conditions of the NVT equilibration run. Each GS system studied was simulated for 10 ns, with B-factor values sampled every 500 MD steps and a maximum MC move of 0.05 nm^2^. To optimize performance, the EMMIVox hybrid energy was calculated every 4 MD timesteps. After single-structure refinement, the conformation with the lowest EMMIVox hybrid energy and the associated B-factor values were extracted. This structure was then energy minimized while the B-factor values were simultaneously sampled using a modified MC sampler allowing only downhill moves. Any discontinuities in the final structure obtained from the energy minimization due to the PBC were then fixed using the PLUMED *driver* tool. We then generated a PDB of the final refined structure and optimized B-factor values. These models represent the final refined single-structure models of each GS system.

EMMIVox ensemble simulations were performed using the same settings as in the single-structure refinement, with the exception of the B-factors, which were fixed, for all residues, at the lowest value obtained from single-structure refinement as a measure of background noise. 20 metainference replicas were used for each system, with each replica run for 20 ns, resulting in 400 ns of total simulation time per system.

### Pairwise residue distance distributions

The probability distribution of residue-residue distances for selected residue pairs were calculated by taking the minimum distance between any two atoms within the selected residue pair for each conformation present in the structural ensemble extracted from EMMIVox. The calculation was performed for each chain within GS, resulting in 10 residue-residue distances extracted for each conformation in the structural ensemble. Kernel density estimation plots were done using a bandwidth adjustment value equal to 1. Histograms plots were calculated using 150 bins.

### Glutamine synthetase knockout HEK293e cell culture and landing pad clone generation

HEK293E glutamine synthetase knockout cells (Yu, Lee, and Lee 2018) and any derivatives were cultured in Dulbecco’s modified Eagle’s medium (DMEM; high glucose, pyruvate, with glutamine; Gibco) supplemented with 10% dialyzed fetal bovine serum (Gibco), 1x GSEM supplement (Sigma-Aldrich), and Penicillin-Streptomycin (10U Penicillin/10 μg ml^−1^; Gibco). Cells were grown on plates at 37 °C with 5% CO2. For routine passaging, cells were detached using trypsin–EDTA 0.25% (Gibco), resuspended in medium, centrifuged for 5 minutes at 200 × g, resuspended and plated.

Landing pad clones were generated as described previously using dAAVS1-TetBxb1BFP (Matreyek, Stephany, and Fowler 2017; Matreyek et al. 2020). Successful clones were verified by genomic PCR and co-transfection of attB-EGFP and attB-mCherry. Clone 15 passed all validation tests and was used for all following experiments (referred to in Methods as GS-KO-TetBxb1BFP and *HEK293E GLUL*^*−/−*^ in main text). Long-term passaging of GS-KO-TetBxb1BFP was done with the addition of 2 μg ml^−1^ doxycycline (Sigma-Aldrich).

### Recombination of single-variant clones or libraries into the GS-knockout-landing pad cell line

Single-variant clones were recombined into the Tet-on landing pad in GS-KO-TetBxb1BFP cell. Transfection of attB-GS variant 0.5 × 10^6^ cells per well transfected with 1.5 μg attB-plasmid DNA and 1.5 μg pCAG-HA-NLS-coBxb1 using Lipofectamine 3000 (Thermo) per manufacturer recommendations. Two days post-transfection, cell media was refreshed with the addition of 2 μg ml^−1^ doxycycline. Four days post-transfection, cells were selected with media containing 2 μg ml^−1^ doxycycline (Sigma-Aldrich) and 0.25 μg ml^−1^ puromycin (Gibco). Cells were selected for one week under puromycin and the resultant stable cell lines were frozen in 10% DMSO (Sigma) in FBS (Gibco) for future experiments.

### Growth test of wild type GS and disease mutants in GS-KO-TetBxb1BFP

To determine the growth rate of different glutamine synthetase variants in the presence and absence of glutamine, stable *HEK293E GLUL*_*variant*_ cell lines (wild-type, C53A, K52A, R298A, L300A, or P242stop) were plated at low confluence (10,000 cells per well) in a series of identical white, tissue culture treated, 96-well plates (GreinerBio) in DMEM (+glutamine and 10% dialyzed FBS, GSEM, 10U Penicillin, 10 μg ml^−1^ Streptomycin, 2 μg ml^−1^ doxycycline, and 0.25 μg ml^−1^ puromycin) and allowed to adhere to the plate for 4 hours. Subsequently, media was swapped (DMEM with 10% dialyzed FBS, GSEM, 10U Penicillin, 10 μg ml^−1^ Streptomycin, 2 μg ml^−1^ doxycycline, and 0.25 μg ml^−1^ puromycin and with or without glutamine) and a Day ‘0’ cell count time point was measured using the CellTiter-Glo kit (Promega). Following the protocol from the vendor, CellTiter-Glo reagent was mixed 1:1 ratio with cells and incubated for 10 mins at room temperature to lyse and provide a luminescence readout. Luminescence was measured on a Veritas luminometer and converted to cell counts from a standard curve of *HEK293E GLUL*_*wild-type*_.

## Supplementary Material

1

## Data Availability

Cryo-EM structures and atomic models have been deposited in the Electron Microscopy Data Bank (EMDB) and Protein Data Bank (PDB), respectively, with the following accession codes: EMD-70845, PDB: 9OTQ (Apo-Filament); EMD-70841, PDB: 9OTM (Turnover Filament); EMD-70843, PDB: 9OTO (Turnover Decamer); EMD-70842, PDB: 9OTN (ATP bound Filament); EMD-70844, PDB: 9OTP (R298A turnover). EMMIVox ensemble refinement and trajectory files are available on Zenodo (10.5281/zenodo.15742546). All the GROMACS topologies and PLUMED input files needed to reproduce our EMMIVox calculations as well as the final refined single-structure models are available in PLUMED-NEST (www.plumed-nest.org), the public repository of the PLUMED consortium (PLUMED consortium 2019), as plumID:25.017.
