# Supplementary material for: Product-stabilized filamentation by human glutamine synthetase allosterically tunes metabolic activity
Source: bioRxiv. 2025 Jul 6:2025.07.04.663231. Preprint. [Version 1] doi: 10.1101/2025.07.04.663231 (PMC12236511; doi:10.1101/2025.07.04.663231)
Supplement: 1 [file NIHPP2025.07.04.663231V1-supplement-1.pdf]

bioRxiv preprint doi: https://doi.org/10.1101/2025.07.04.663231; this version posted July 6, 2025. The copyright holder for this preprint (which was not certified by peer review) is the author/funder, who has granted bioRxiv a license to display the preprint in perpetuity. It is made available under aCC-BY 4.0 International license.

Supplementary Material

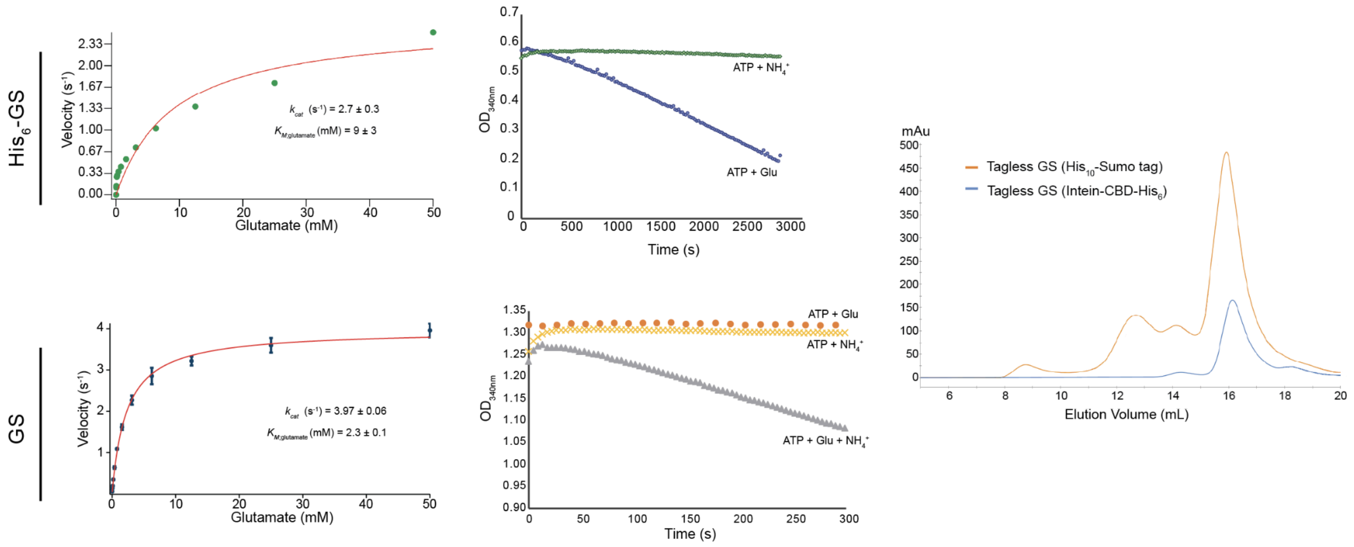

Supplementary Figure 1

Biochemical characterization of recombinant human GS. Left: Steady-state kinetic analysis of N-terminally His_6-tagged GS (top) and tagless GS derived from the GS-Intein-CBD-His_6 tagged construct where the $Km_{(glu)}$ is noted to be 3-fold higher for His_6-tagged GS and His_6-tagged GS is noted to display non-specific ATP hydrolysis under ATP + Glutamate conditions suggesting potential loss of γ-glutaryl phosphate intermediate. Right: size-exclusion chromatography traces of tagless GS constructs derived either from the His_{10}-Sumo tagged GS or Intein-CBD-His_6 tagged GS where in both cases the predominant molecular weight species is found at ~16 mL elution volume on a superose6 increase (Cytiva) column which corresponds to the decameric species.

bioRxiv preprint doi: https://doi.org/10.1101/2025.07.04.663231; this version posted July 6, 2025. The copyright holder for this preprint (which was not certified by peer review) is the author/funder, who has granted bioRxiv a license to display the preprint in perpetuity. It is made available under aCC-BY 4.0 International license.

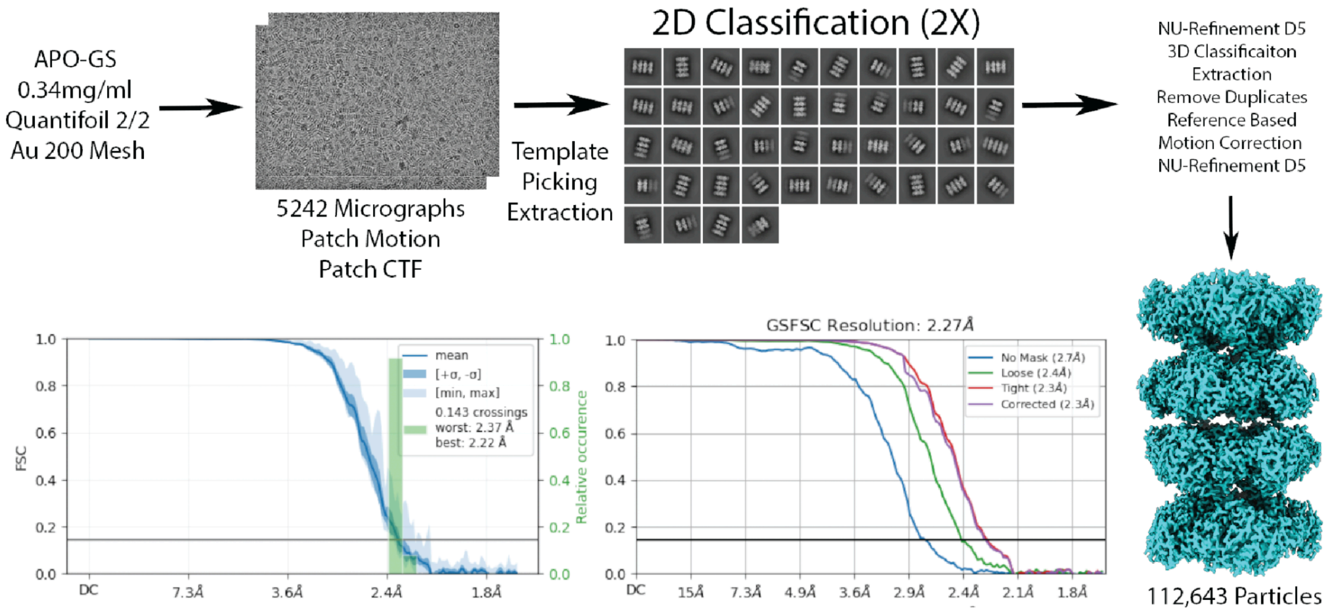

Supplementary Figure 2

cryoEM data processing pipeline for apo-filament map. Depicted in the pipeline includes grid type, protein concentration, representative micrographs, utilized 2D classes, 3D reconstruction details, and final maps with associated particle counts and FSC curves.

bioRxiv preprint doi: https://doi.org/10.1101/2025.07.04.663231; this version posted July 6, 2025. The copyright holder for this preprint (which was not certified by peer review) is the author/funder, who has granted bioRxiv a license to display the preprint in perpetuity. It is made available under aCC-BY 4.0 International license.

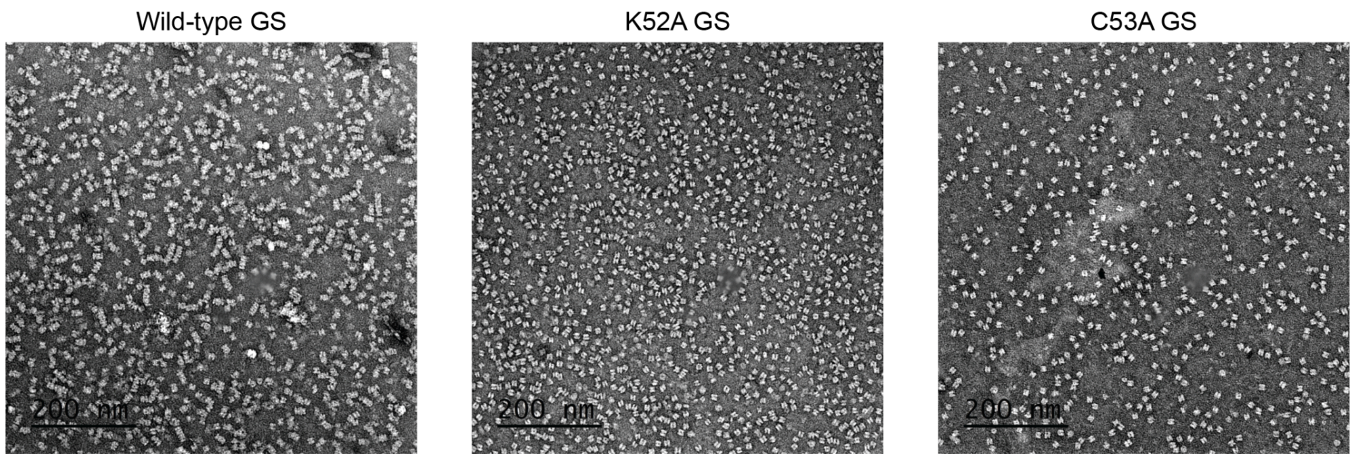

Supplementary Figure 3.

Negative stain EM of human glutamine synthetase variants showing attenuation of filament formation for the point mutants K52A (middle) and C53A (right) compared to wild-type (left).

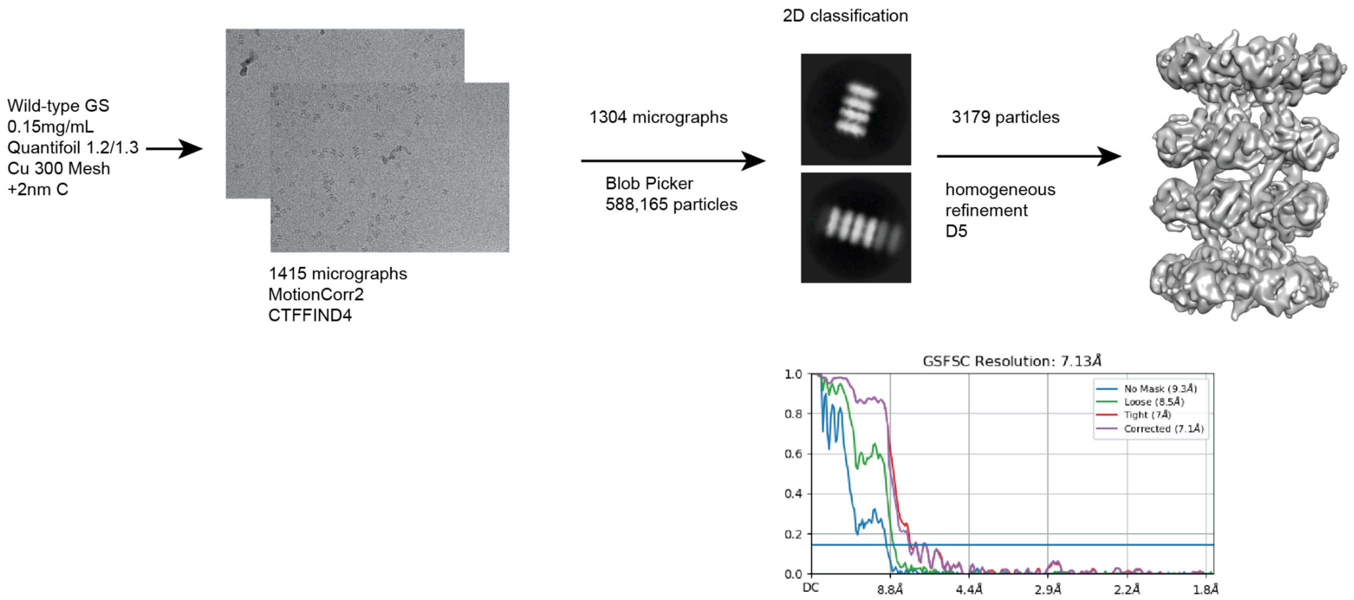

Supplementary Figure 4

cryoEM data processing pipeline for initial low resolution turnover-filament. cryoEM data processing pipeline for low resolution turnover filament map. Depicted in the pipeline includes grid type, protein

bioRxiv preprint doi: https://doi.org/10.1101/2025.07.04.663231; this version posted July 6, 2025. The copyright holder for this preprint (which was not certified by peer review) is the author/funder, who has granted bioRxiv a license to display the preprint in perpetuity. It is made available under aCC-BY 4.0 International license.

concentration, representative micrographs, utilized 2D classes, 3D reconstruction details, and final map with FSC curve.

bioRxiv preprint doi: https://doi.org/10.1101/2025.07.04.663231; this version posted July 6, 2025. The copyright holder for this preprint (which was not certified by peer review) is the author/funder, who has granted bioRxiv a license to display the preprint in perpetuity. It is made available under aCC-BY 4.0 International license.

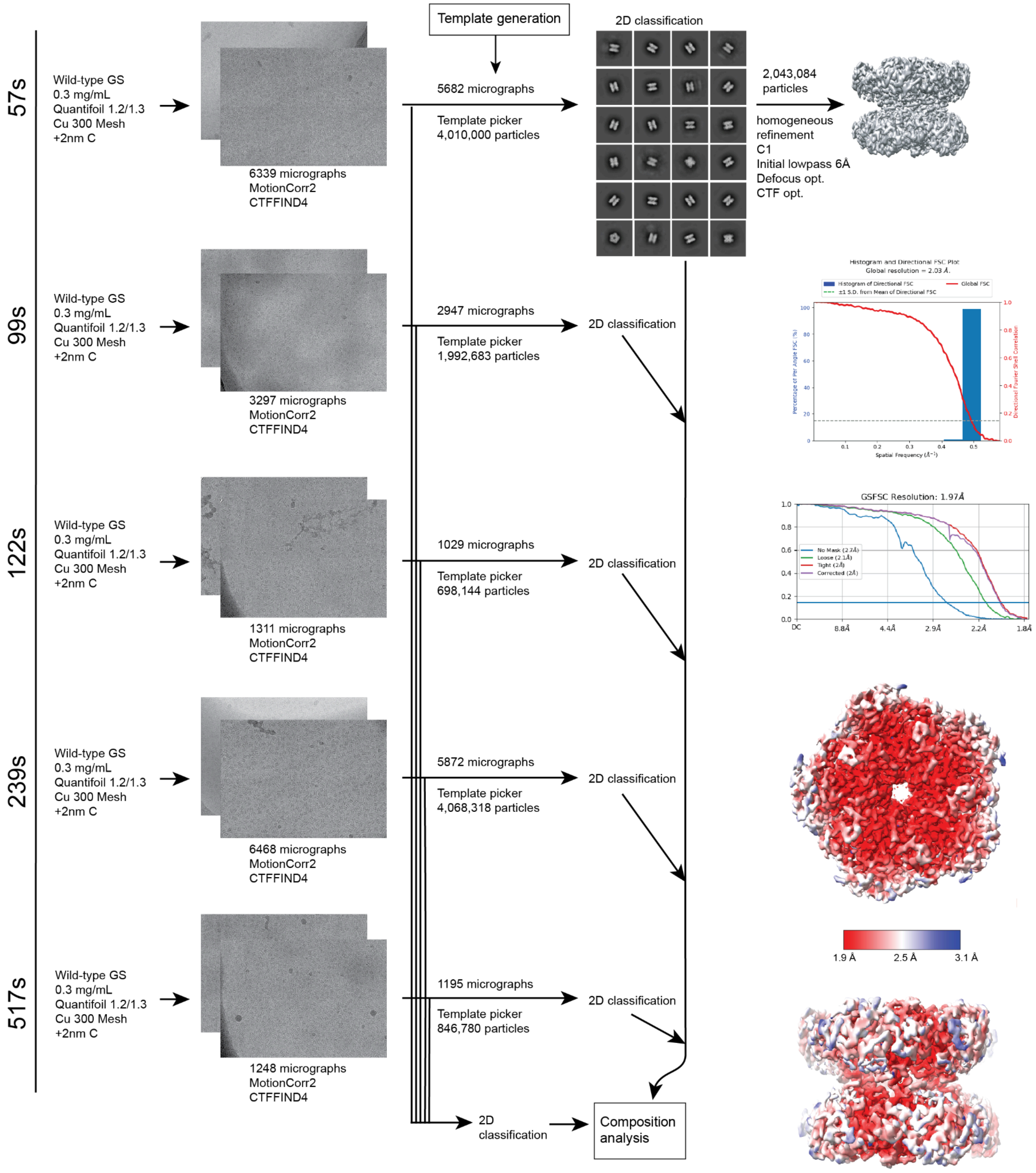

bioRxiv preprint doi: https://doi.org/10.1101/2025.07.04.663231; this version posted July 6, 2025. The copyright holder for this preprint (which was not certified by peer review) is the author/funder, who has granted bioRxiv a license to display the preprint in perpetuity. It is made available under aCC-BY 4.0 International license.

Supplementary Figure 5

cryoEM data processing pipeline for time-resolved datasets and turnover-decamer map. Depicted in the pipeline includes five total datasets where in each dataset, the grid type, protein concentration, representative micrographs, and 2D processing workflow. In addition, the 57s turnover dataset was for 3D reconstruction of the decamer turnover map and displays the utilized 2D classes, 3D reconstruction details, and final maps with associated particle counts, local resolution estimation maps, and FSC curves.

bioRxiv preprint doi: https://doi.org/10.1101/2025.07.04.663231; this version posted July 6, 2025. The copyright holder for this preprint (which was not certified by peer review) is the author/funder, who has granted bioRxiv a license to display the preprint in perpetuity. It is made available under aCC-BY 4.0 International license.

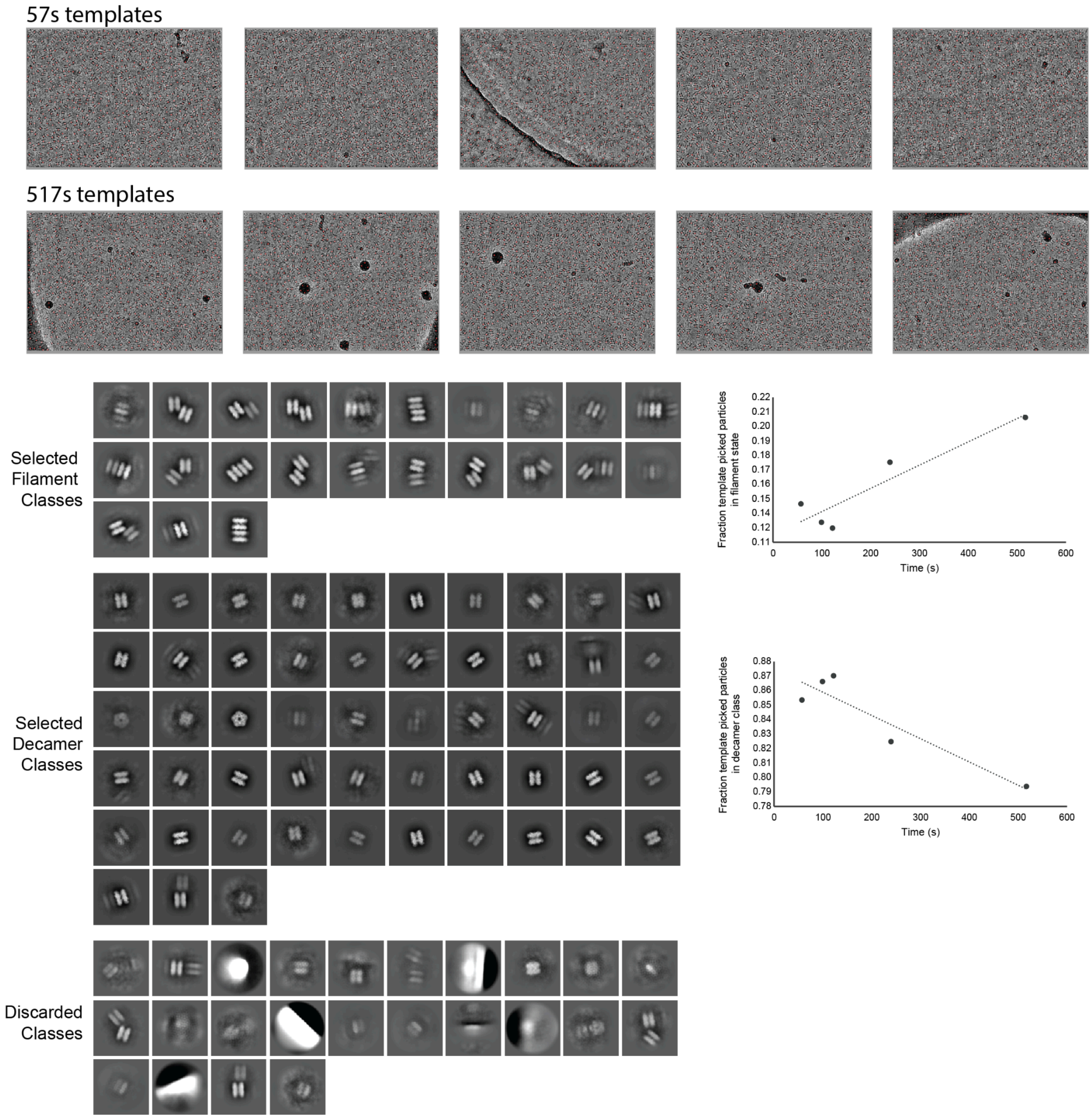

Supplementary Figure 6

Global 2D classification of time-resolved datasets. Representative template picked particle images for the 57s and 517s datasets are depicted above. All five tr-cryoEM datasets were combined and classified in two dimensions and separated into filament, decamer, and discarded classes with 2D class images depicted at bottom left. 2D classes where partial head-on-head contacts could be discerned were included in the filament classification. 2D classes where multiple decamers were present, but not in a head-on-head arrangement were discarded. The filament and decamer particles

bioRxiv preprint doi: https://doi.org/10.1101/2025.07.04.663231; this version posted July 6, 2025. The copyright holder for this preprint (which was not certified by peer review) is the author/funder, who has granted bioRxiv a license to display the preprint in perpetuity. It is made available under aCC-BY 4.0 International license.

classes were then referenced to origin dataset using cryoSPARC tools and the fraction of each species was quantified and linearly fit (bottom right).

bioRxiv preprint doi: https://doi.org/10.1101/2025.07.04.663231; this version posted July 6, 2025. The copyright holder for this preprint (which was not certified by peer review) is the author/funder, who has granted bioRxiv a license to display the preprint in perpetuity. It is made available under aCC-BY 4.0 International license.

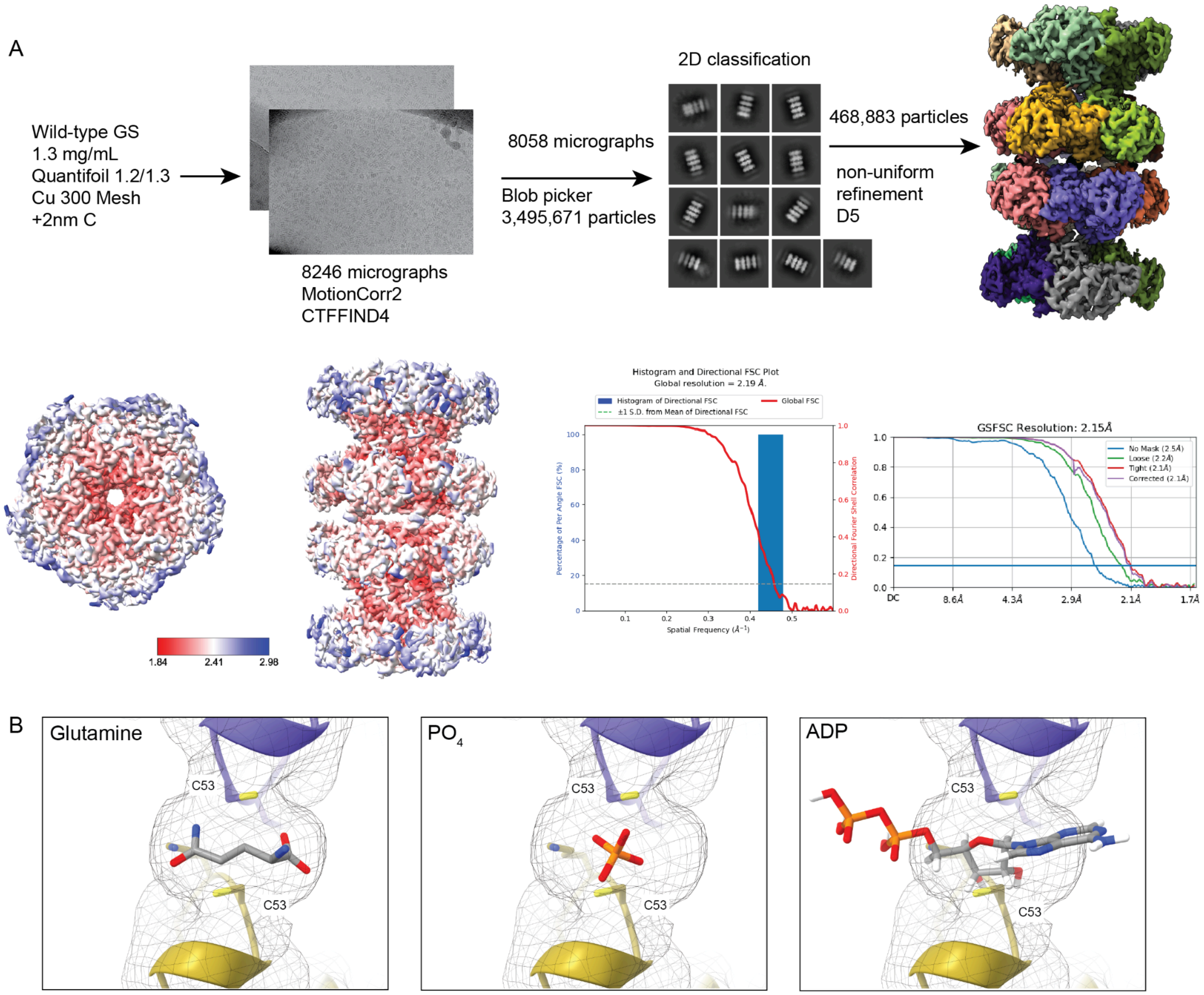

Supplementary Figure 7

A. CryoEM data processing pipeline for high resolution turnover-filament. Depicted in the pipeline includes grid type, protein concentration, representative micrographs, utilized 2D classes, 3D reconstruction details, and final maps with associated particle counts. Below are local resolution estimation maps and FSC curves for the final map. B. GS products (glutamine, PO_4, and ADP) individually fit into filament interface density.

bioRxiv preprint doi: https://doi.org/10.1101/2025.07.04.663231; this version posted July 6, 2025. The copyright holder for this preprint (which was not certified by peer review) is the author/funder, who has granted bioRxiv a license to display the preprint in perpetuity. It is made available under aCC-BY 4.0 International license.

bioRxiv preprint doi: https://doi.org/10.1101/2025.07.04.663231; this version posted July 6, 2025. The copyright holder for this preprint (which was not certified by peer review) is the author/funder, who has granted bioRxiv a license to display the preprint in perpetuity. It is made available under aCC-BY 4.0 International license.

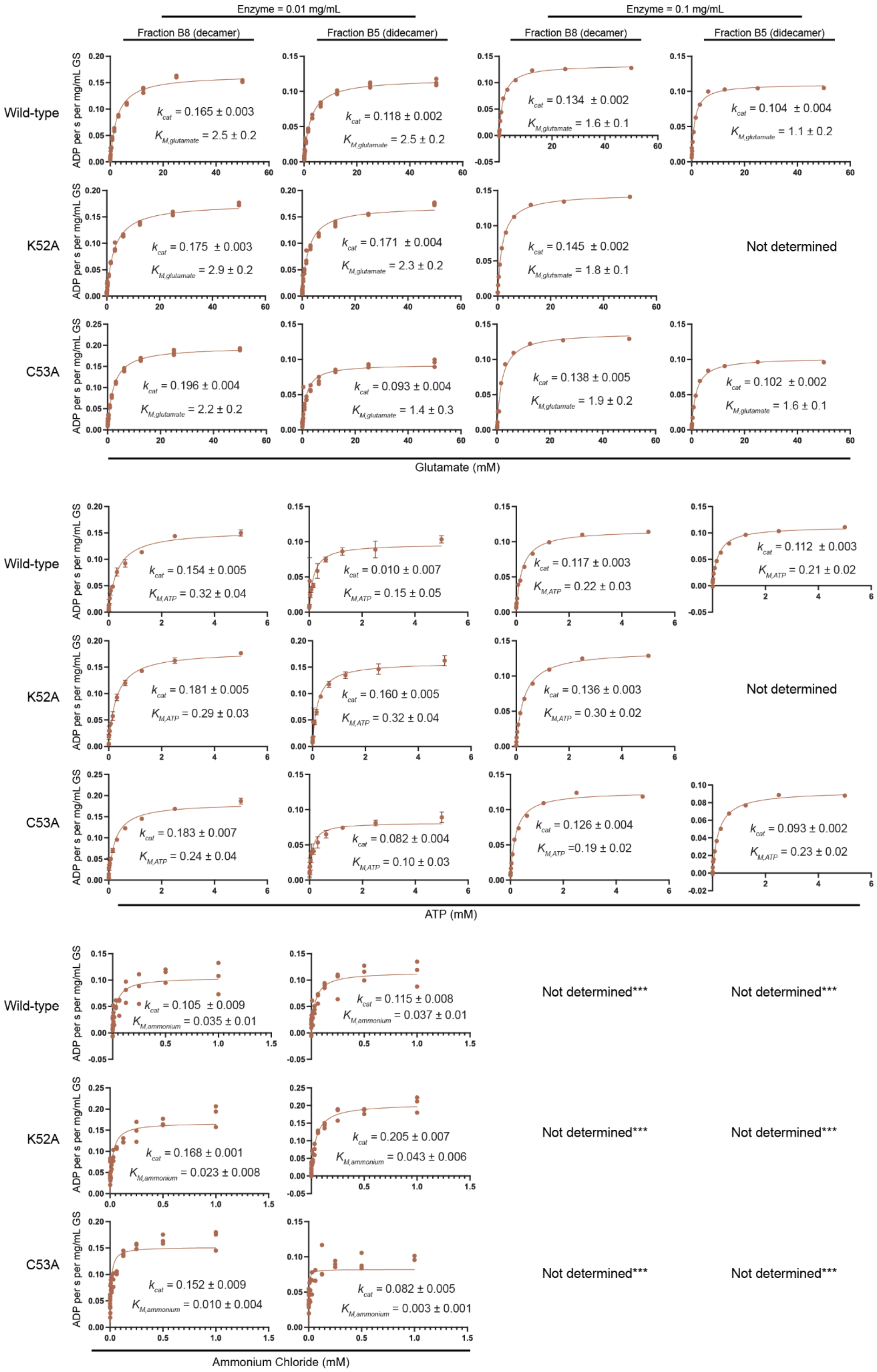

bioRxiv preprint doi: https://doi.org/10.1101/2025.07.04.663231; this version posted July 6, 2025. The copyright holder for this preprint (which was not certified by peer review) is the author/funder, who has granted bioRxiv a license to display the preprint in perpetuity. It is made available under aCC-BY 4.0 International license.

Supplementary Figure 8

Steady-state kinetic screening with Michaelis-Menten fitting for wild-type, K52A, and C53A GS variants. Initial velocities from the coupled-ATPase assay measurements are presented with fits to a basic Michaelis-Menten model included. Error was estimated from goodness of fit as S.E.M. Enzyme was assayed at 0.01 mg/mL (left) or 0.1 mg/mL (right) and sample from either the B8 (decamer) or B5 (2-decamer) fraction from size-exclusion chromatography from a Sup6 Increase column. The K52A and C53A variants displayed nearly identical Michaelis-Menten parameters as wild-type under both conditions. K52A B5 fraction was not of high enough concentration to assay at 0.1 mg/mL and thus not determined. *** indicates that the rate of conversion at 0.1 mg/mL enzyme was too fast to capture by initial velocity analysis.

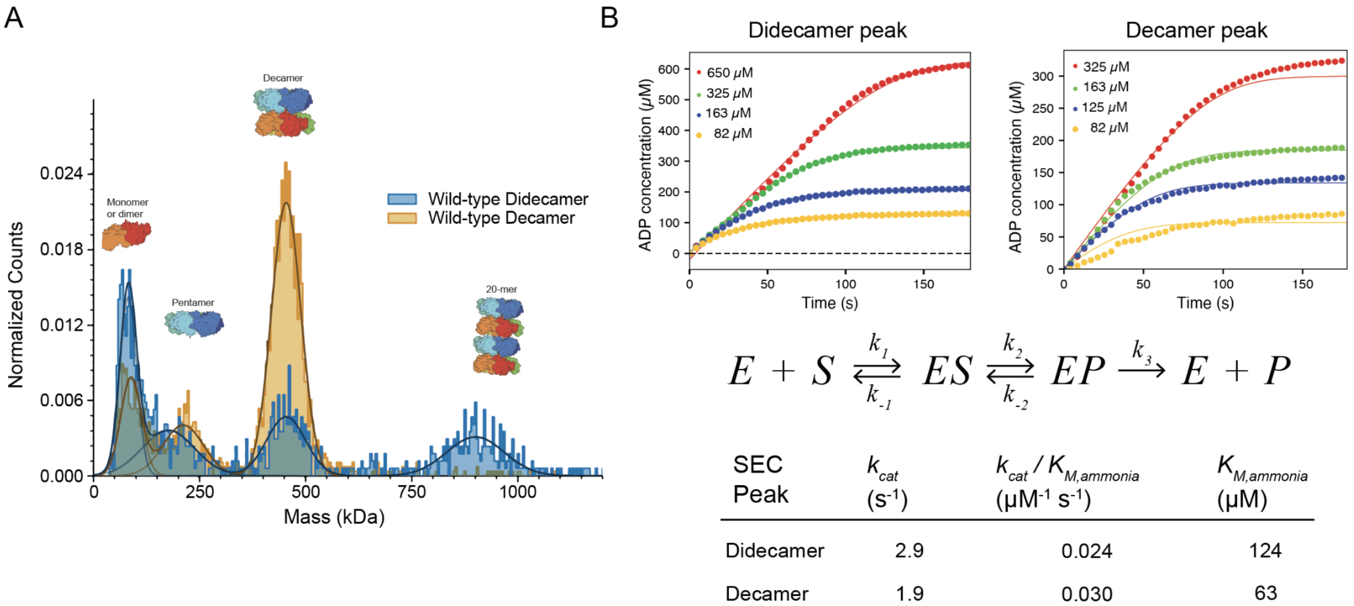

$$E + S \underset{k_{-1}}{\overset{k_1}{\rightleftharpoons}} ES \underset{k_{-2}}{\overset{k_2}{\rightleftharpoons}} EP \xrightarrow{k_3} E + P$$

SEC Peak	k_{cat} (s^{-1})	$k_{cat} / K_{M,ammonia}$ ($\mu M^{-1} s^{-1}$)	$K_{M,ammonia}$ (μM)
Didecamer	2.9	0.024	124
Decamer	1.9	0.030	63

Supplementary Figure 9

Initial assessment of filament steady-state kinetics. A) mass photometry assessment of oligomeric state for wild-type GS at 0.01 mg/mL demonstrating presence of 2-decameric enzyme in the 2-decameric SEC peak but not in the decameric SEC peak fraction. Additionally observed is the multiple oligomeric states GS could be found in solution at lower concentration. B) Steady-state product formation data for wild-type GS 2-decameric or decameric SEC peaks performed at 0.1 mg/mL GS with varied ammonia concentrations. Top, ADP product concentration (n=1 per concentration) per time at multiple ammonia concentrations with saturating glutamate and ATP globally fit to a simple Michaelis-Menten Model shown in middle using Kintek Explorer. Fit parameters displayed at bottom demonstrate a 2-fold change in $K_{M,ammonia}$. An approximate sigma value of 30 μM was used for fitting each dataset.

bioRxiv preprint doi: https://doi.org/10.1101/2025.07.04.663231; this version posted July 6, 2025. The copyright holder for this preprint (which was not certified by peer review) is the author/funder, who has granted bioRxiv a license to display the preprint in perpetuity. It is made available under aCC-BY 4.0 International license.

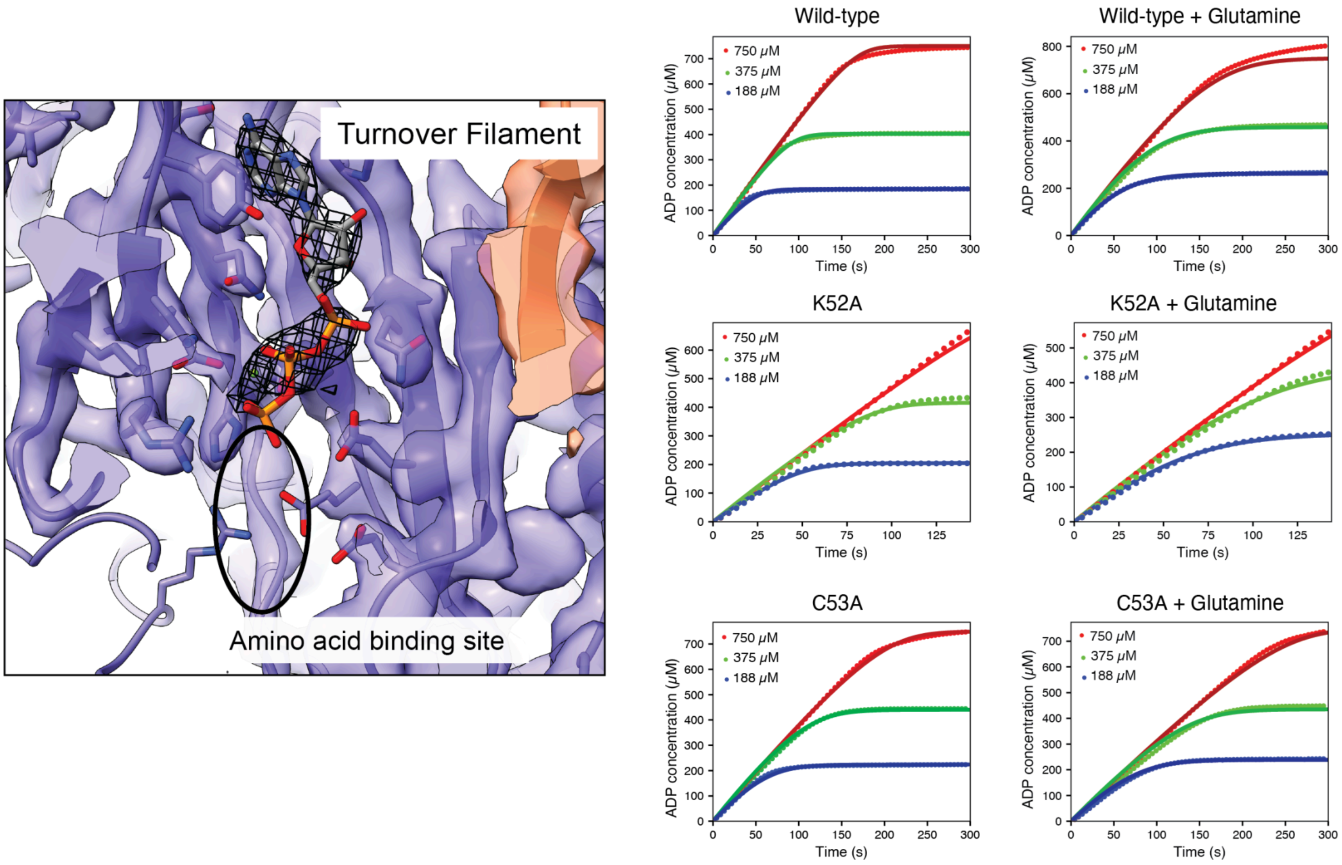

Supplementary Figure 10

Glutamine-stabilized filament steady-state global fitting to determine $K_{M,ammonia}$. The turnover filament map is presented in purple (chain A), black mesh (ligand of chain A), and orange (chain E). Active site residues are shown from the consensus EMMIVox model. Absence of density corresponding to the amino acid binding pocket is shown (black circle) demonstrating that despite high glutamine levels in this dataset, no glutamine is bound to the active site. Right, steady-state data is presented as an average of eight independent progress curve datasets with GS at 0.1 mg/mL, glutamate at 50 mM starting concentration, ATP at 5 mM starting concentration, and ammonium chloride at 0.75, 0.375, and 0.188 mM as indicated. Data were globally fit to a simple Michael-Menten model (bottom left) using Kintek Explorer. Fits are included in plotted data. An average sigma value of 50 μM was used for fitting each dataset.

bioRxiv preprint doi: https://doi.org/10.1101/2025.07.04.663231; this version posted July 6, 2025. The copyright holder for this preprint (which was not certified by peer review) is the author/funder, who has granted bioRxiv a license to display the preprint in perpetuity. It is made available under aCC-BY 4.0 International license.

	Chain A	Atoms outside contour Residues 265-316	Chain G	Atoms outside contour Residues 265-316
Apo-Filament	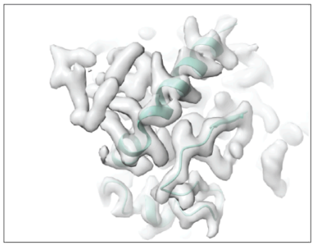	468	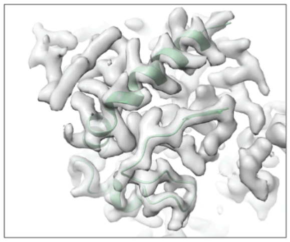	407
ATP Filament	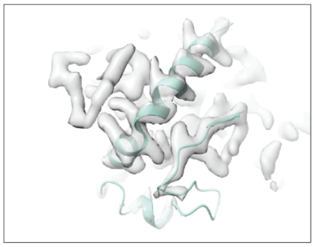	554	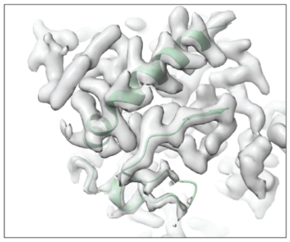	448
Turnover Decamer	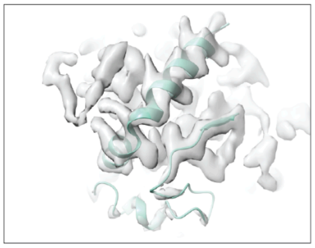	598	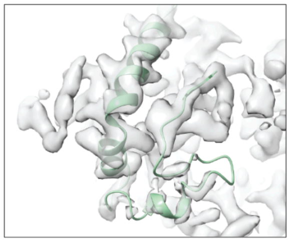	560
Turnover Filament	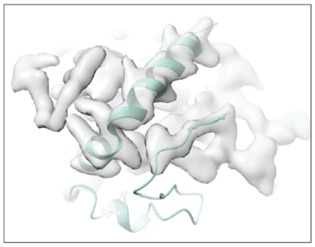	570	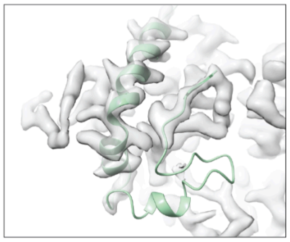	613

Supplementary Figure 11.

Relative loop density correlations between cryoEM maps. Comparison of cryoEM density maps between apo-filament, ATP filament, Turnover Decamer and Turnover Filament for Chains A and G across the pentamer:pentamer interface. Density was manually thresholded in ChimeraX to be approximately similar by side change density for residues in helix 8 (265-285). A subsegment of PDB 2QC8 (residues 265-316 which includes the E305-loop (residues 289-309)) was fit into the density using the ‘Fit in Map’ tool in ChimeraX and the residues outside the contour level are reported. In general, the apo-filament map had the most density corresponding to the E305-loop, followed by the ATP filament map, Turnover Decamer map, and Turnover Filament map.

bioRxiv preprint doi: https://doi.org/10.1101/2025.07.04.663231; this version posted July 6, 2025. The copyright holder for this preprint (which was not certified by peer review) is the author/funder, who has granted bioRxiv a license to display the preprint in perpetuity. It is made available under aCC-BY 4.0 International license.

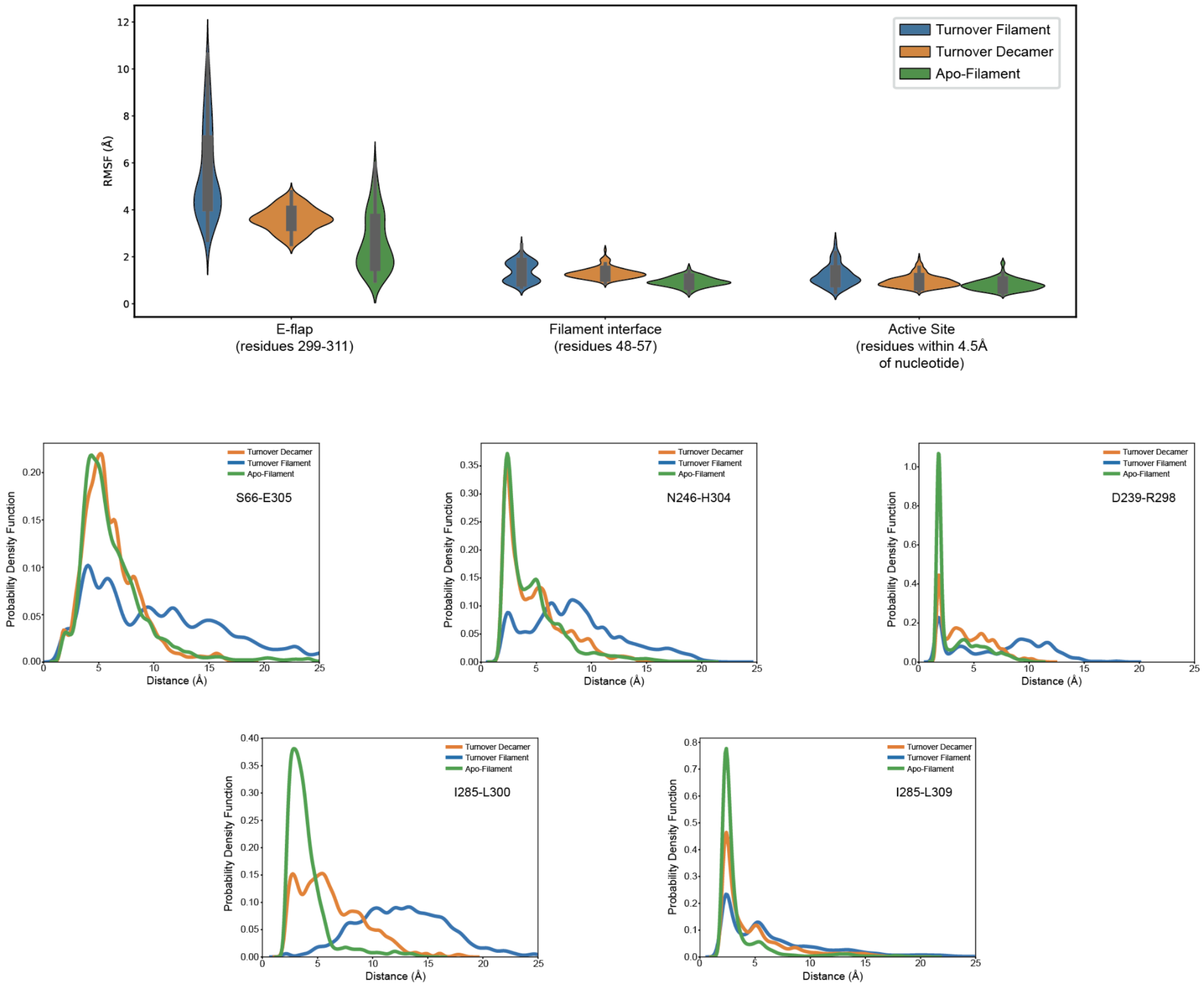

Supplementary Figure 12

Ensemble analysis including apo-filament dataset. Top: RMSF violin plots depicting root mean square fluctuation (RMSF) of E305-loop (residues 299-311), Filament Interface loop (residues 48-57), or ‘top’ of bifunnel active site (residues within 4.5 Å of bound nucleotide) showing highest fluctuation for the E305-loop and within the E305-loop region, the highest RMSF is noted for the turnover filament followed by turnover decamer and these least RMSF for the apo-filament dataset. Both the filament interface loop and active site displayed low RMSF overall. Bottom: Similar to Figure 3 but including overlay of apo-filament ensemble data. MD-ensemble refinement distance quantification of S66-E305, N246-H304, D239-R298, I285-L300, and I285-L309. In all cases, the apo ensemble demonstrates a more stable E305-loop where all stabilizing interactions are maintained with higher probability.

bioRxiv preprint doi: https://doi.org/10.1101/2025.07.04.663231; this version posted July 6, 2025. The copyright holder for this preprint (which was not certified by peer review) is the author/funder, who has granted bioRxiv a license to display the preprint in perpetuity. It is made available under aCC-BY 4.0 International license.

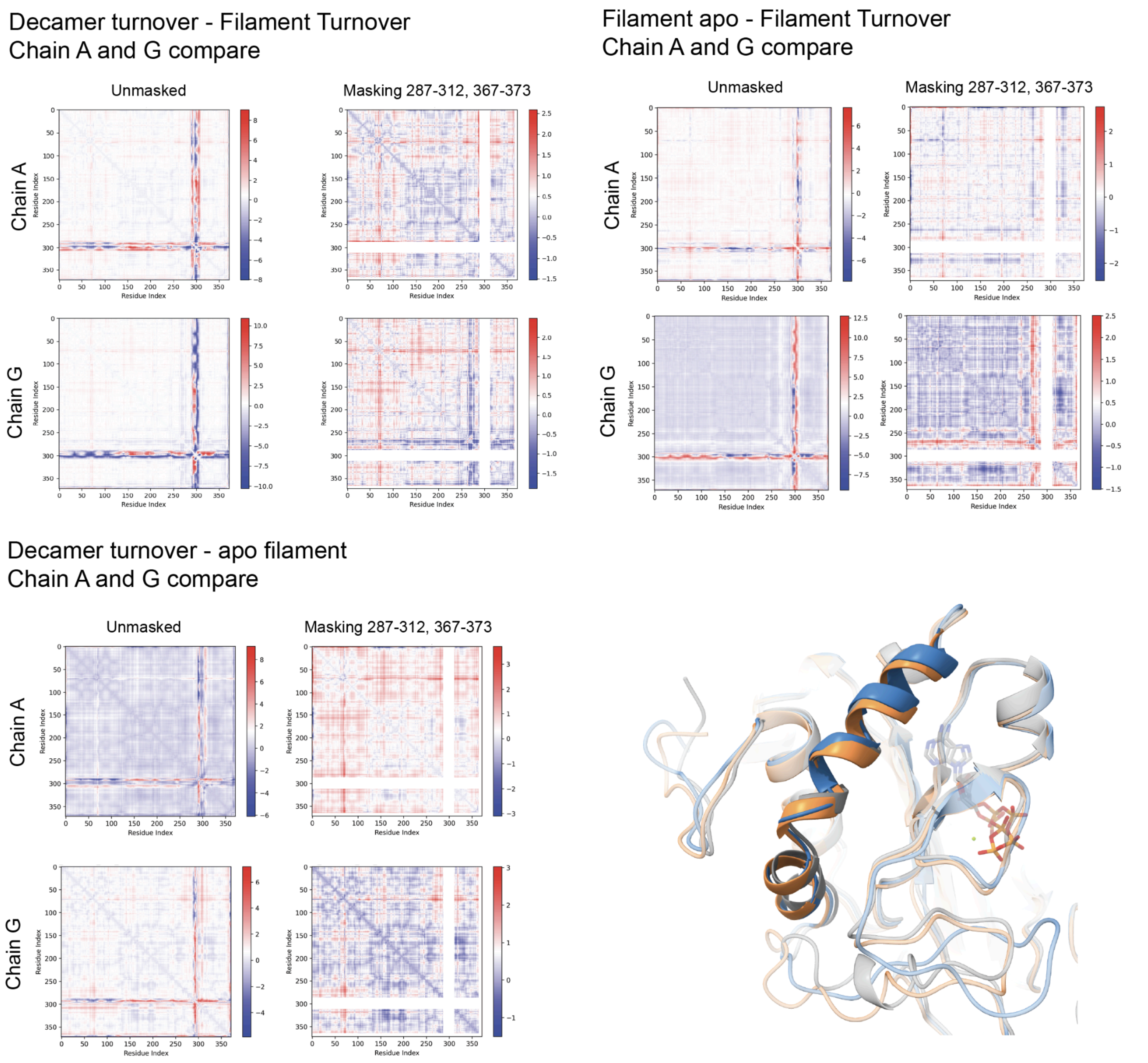

Supplementary Figure 13

Global conformational changes most apparent in Turnover Filament structure. C-α distance difference matrix for chains A and G between Turnover Decamer and Turnover Filament models (top left), Apo Filament and Turnover- Filament models (top right), and Decamer Turnover and Apo Filament models (bottom left) depicted with and without the E305-loop (residues 289-312) and the far C-terminal peptide (369-373) omitted. Absolute distance color legend at right of each plot is in units of Å. These comparisons demonstrate that the tip of the B-grasp region (60-76) and helix 8 (residues 262-282) display conformational differences for the Filament Turnover model primarily. Bottom right: overlay of

bioRxiv preprint doi: https://doi.org/10.1101/2025.07.04.663231; this version posted July 6, 2025. The copyright holder for this preprint (which was not certified by peer review) is the author/funder, who has granted bioRxiv a license to display the preprint in perpetuity. It is made available under aCC-BY 4.0 International license.

Turnover Filament (blue), Turnover Decamer (orange), and Apo Filament (gray) models highlighting helix 8 (residues 262-282) and showing a loss of helicity in part of helix 8 for Turnover Filament only.

bioRxiv preprint doi: https://doi.org/10.1101/2025.07.04.663231; this version posted July 6, 2025. The copyright holder for this preprint (which was not certified by peer review) is the author/funder, who has granted bioRxiv a license to display the preprint in perpetuity. It is made available under aCC-BY 4.0 International license.

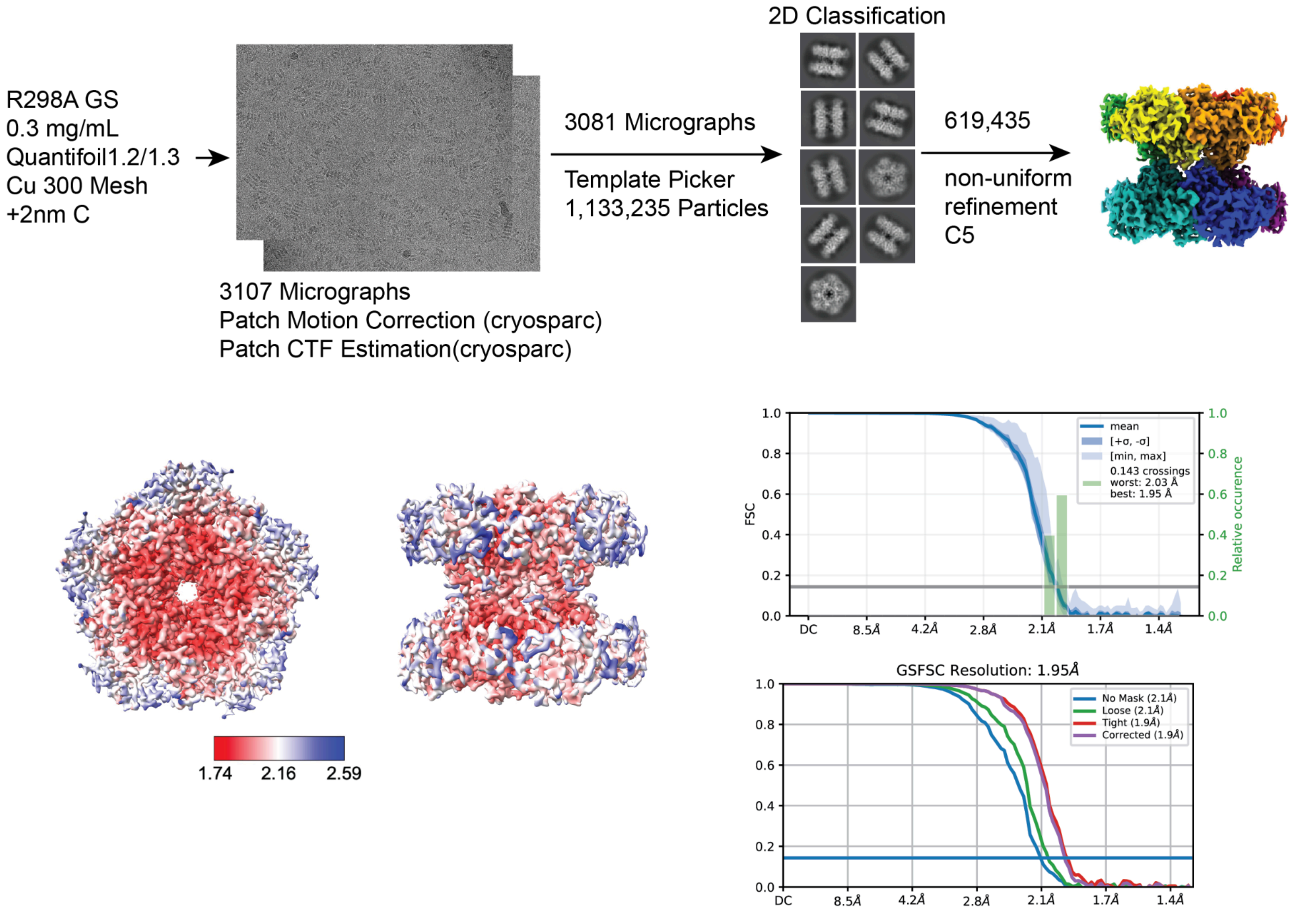

Supplementary Figure 14

cryoEM processing pipeline for R298A turnover-decamer and comparison to wild-type turnover decamer. Depicted in the pipeline includes grid type, protein concentration, representative micrographs, utilized 2D classes, 3D reconstruction details, and final maps with associated particle counts. At the bottom left are local resolution estimation maps and FSC curves for the final map.

bioRxiv preprint doi: https://doi.org/10.1101/2025.07.04.663231; this version posted July 6, 2025. The copyright holder for this preprint (which was not certified by peer review) is the author/funder, who has granted bioRxiv a license to display the preprint in perpetuity. It is made available under aCC-BY 4.0 International license.

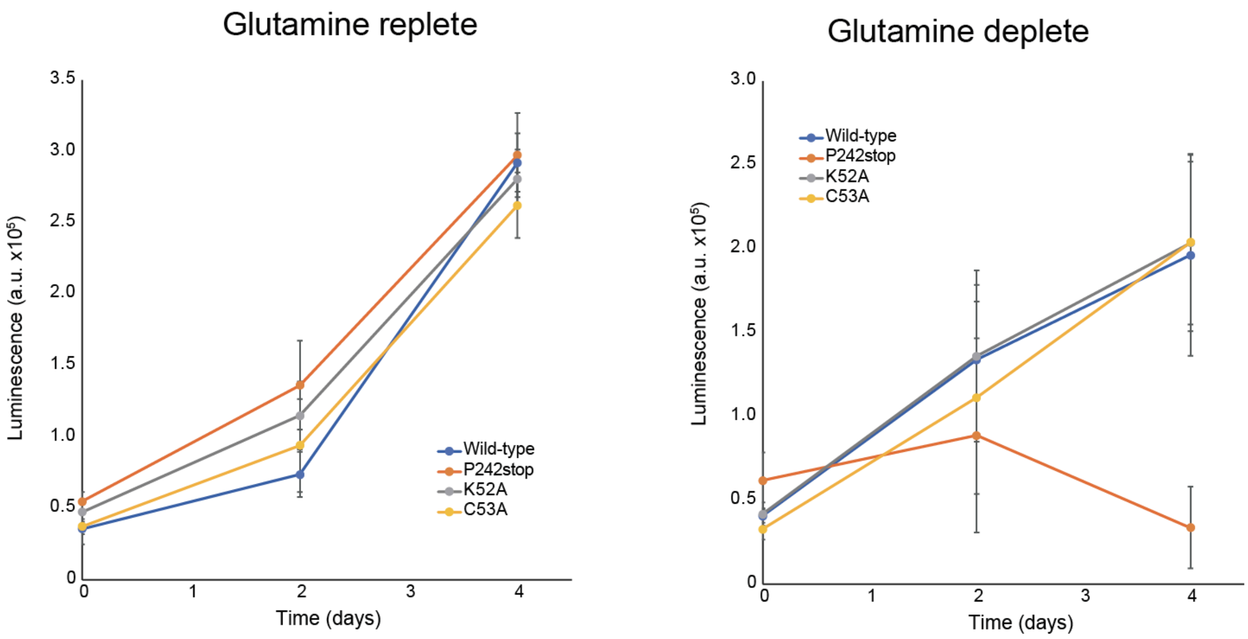

Supplementary Figure 15

Glutamine auxotrophy determination of filament interface mutants. Luminescence derived from Promega CellTiter Glo plotted against growth time for wild-type, C53A, K52A, and P242stop variants under glutamine replete (left) or deplete (right) media conditions demonstrating similar GS-dependent growth rates for wild-type, C53A, and K52A variants, while P242stop GS could not support cell growth.

bioRxiv preprint doi: https://doi.org/10.1101/2025.07.04.663231; this version posted July 6, 2025. The copyright holder for this preprint (which was not certified by peer review) is the author/funder, who has granted bioRxiv a license to display the preprint in perpetuity. It is made available under aCC-BY 4.0 International license.

Supplementary Table1

Structure	Apo Filament	Turnover Filament	Turnover Decamer	ATP Filament	R298A decamer
Ligands	Mg(II)	ADP, Mg(II)	ADP, Mg(II)	ATP, Mg(II)	ADP, Mg(II)
PDB code	9OTQ	9OTM	9OTO	9OTN	9OTP
EMDB code	EMD-70845	EMD-70841	EMD-70843	EMD-70842	EMD-70844
Magnification	105,000	106,000	106,000	105,000	134,000
Voltage (kV)	300	300	300	300	300
Electron fluence ($e^-/Å^2$)	60	45	40	64.2	40
Defocus range (µm)	0.8-1.8	0.6-2.0	0.6-2.0	0.8-1.8	0.6-2.0
Pixel size (data collection) (Å)	0.4215	0.82	0.86	0.4215	0.664
Pixel size (reconstruction) (Å)	0.843	0.82	0.86	0.843	0.664
Symmetry imposed	D5	D5	C1	D5	C5
Particle images (no.)	112,643	468,883	2,043,084	310,500	619,435
Resolution (0.143 FSC) (Å)	2.27	2.19	2.03	2.11	1.95
R.m.s. deviation	0.004	0.004	0.004	0.004	0.04

bioRxiv preprint doi: https://doi.org/10.1101/2025.07.04.663231; this version posted July 6, 2025. The copyright holder for this preprint (which was not certified by peer review) is the author/funder, who has granted bioRxiv a license to display the preprint in perpetuity. It is made available under aCC-BY 4.0 International license.

**bond lengths (Å)**					
**R.m.s. deviation bond angles (°)**	0.942	0.925	1.018	0.933	1.026
**MolProbity score**	1.02	1.13	1.42	0.96	1.26
**Clashscore**	2.36	3.42	2.95	1.92	3.11
**C-beta outliers (%)**	0.00	0.00	0.00	0.00	0.00
**Rotamer outliers (%)**	0.14	0.14	1.15	0.32	0.55
**Ramachandran favored (%)**	98	98	96	98	97
**Ramachandran allowed (%)**	2	2	4	2	3
**Ramachandran outliers (%)**	0	0	0	0	0
